# Sensor-based intervention to enhance movement control of the spine in low back pain: Protocol for a quasi-randomized controlled trial

**DOI:** 10.3389/fspor.2022.1010054

**Published:** 2022-10-17

**Authors:** Bianca M. P. Mourits, Lammert A. Vos, Sjoerd M. Bruijn, Jaap H. van Dieën, Maarten R. Prins

**Affiliations:** ^1^Research and Development, Military Rehabilitation Center “Aardenburg”, Doorn, Netherlands; ^2^Department of Human Movement Sciences, Amsterdam Movement Sciences, Vrije Universiteit Amsterdam, Amsterdam, Netherlands; ^3^Faculty of Behavioural and Movement Sciences, Institute of Brain and Behavior Amsterdam, Vrije Universiteit Amsterdam, Amsterdam, Netherlands; ^4^Institute for Human Movement Studies, HU University of Applied Sciences Utrecht, Utrecht, Netherlands

**Keywords:** low back pain, movement control, Static motor control, spine, exergaming, rehabilitation

## Abstract

**Introduction:**

Chronic low back pain is a common condition that imposes an enormous burden on individuals and society. Physical exercise with education is the most effective treatment, but generally results in small, albeit significant improvements. However, which type of exercise is most effective remains unknown. Core stability training is often used to improve muscle strength and spinal stability in these patients. The majority of the core stability exercises mentioned in intervention studies involve no spinal movements (static motor control exercises). It is questionable if these exercises would improve controlled movements of the spine. Sensor-based exergames controlled with spinal movements could help improve movement control of the spine. The primary aim of this study is to compare the effects of such sensor-based exergames to static motor control exercises on spinal movement control.

**Methods and analysis:**

In this quasi-randomized controlled trial, 60 patients with chronic low back pain who are already enrolled in a multidisciplinary rehabilitation programme will be recruited. Patients will be randomly allocated into one of two groups: the Sensor-Based Movement Control group (*n* = 30) or the Static Motor Control group (*n* = 30). Both groups will receive 8 weeks of two supervised therapy sessions and four home exercises per week in addition to the rehabilitation programme. At baseline (week 1) and after the intervention (week 10), movement control of the spine will be assessed using a tracking task and clinical movement control test battery. Questionnaires on pain, disability, fear avoidance and quality of life will be taken at baseline, after intervention and at 6- and 12 months follow-up. Repeated measures ANOVAs will be used to evaluate if a significant Group x Time interaction effect exists for the movement control evaluations.

**Discussion:**

Sensor-based spinal controlled exergames are a novel way to train spinal movement control using meaningful and engaging feedback. The results of this study will inform clinicians and researchers on the efficacy of movement control training for patients with low back pain.

**Ethics and dissemination:**

Ethical approval for this study protocol was obtained from the METC Brabant (protocol number NL76811.028.21).

**Trial registration:**

Open Science Framework Registries (https://osf.io/v3mw9/), registration number: 10.17605/OSF.IO/V3MW9, registered on 1 September 2021.

## Introduction

Low back pain is extremely common. More than 50% of the population will experience one or more episodes of low back pain during their lifetime (1). Most episodes of low back pain resolve within 6 weeks, but in some cases the symptoms return regularly. In those cases where symptoms persist for more than 3 months, there is a chronic condition with a variable course (2, 3). According to current evidence, the best treatment for low back pain is exercise, preferably in combination with education, but thus far, to the best of our knowledge there is no evidence that certain exercises work better than others (4).

In a considerable part of the intervention studies with a focus on physical exercises, “core stability” interventions are offered (5). During these interventions, patients are taught to selectively contract the deep trunk muscles (m. Transversus Abdominis and mm. Multifidi) in various postures and during various movements of the extremities. The lumbar spine is fixated in neutral lordosis in most of these exercises (6). We will refer to this type of exercise as “static motor control exercise” henceforth. Although these exercises have been shown to be effective in pain reduction, they are not superior to other physical exercise interventions (6).

Some patients with low back pain fixate their spine (i.e., they demonstrate reduced range of motion) during every-day movements (7–11). This behavior could be stimulated further with static motor control exercises. Moreover, several low back pain patients do experience problems with spinal movement control (12), i.e., adapting the direction, speed, and amplitude of spinal movement to the demands of the task at hand.

Designing exercises to improve movement control of the spine is a challenge. In a recent paper by Hooker et al., patients with low back pain received patient specific training to modify their altered movement pattern during functional activities (13). This resulted in a more normal distribution of hip, knee and spinal movements when picking up an object at shank height. Although this study shows that training can improve the relative contribution of joint movements during functional tasks in low back pain patients, it is no direct evidence that spinal movement control has improved.

Movement control over less centrally located joints, such as the elbow or knee, can be trained using functional tasks, like bringing a spoon to the mouth or kicking a ball toward a pylon. The success of the execution (not spilling the soup or knocking over the pylon) can be used as an indication of good control over the movement of the joint. Providing meaningful feedback on spinal movements is more complicated. Sensors that measure spinal movements can offer a solution (14). There are several sensor-based training systems available on the market, but currently only a few randomized controlled trials incorporating these technologies have been published (15, 16). These sensor-based training systems can be used to offer accurate real-time feedback on spinal movements, which could help to improve spinal movement control. These systems provide the possibility to train spinal movement control relatively independent without the need of intensive supervision and/or a highly experienced therapist (17). Moreover, the training sessions are relatively easy to standardize and the progression from simple toward complex movements can easily be adapted to each patient's abilities and needs. The sensor-based exergames could be more engaging and motivating than conventional motor controlexercises, which might increase therapy adherence (18).

This paper describes the protocol for a randomized controlled trial to evaluate if a sensor-based movement control intervention enhances movement control of the spine in low back pain patients to a greater extent than a standard static motor control intervention. We will assess movement control using a custom made spinal movement controlled tracking task and a clinical test battery by Luomajoki et al. (19, 20). We hypothesize that a sensor-based movement control intervention will enhance movement control of the spine in low back pain patients measured by spinal movement controlled tracking tasks to a greater extent than a standard static motor control intervention. The majority of the tests in the clinical test battery by Luomajoki et al. (19, 20) involve no spinal movement, hence we hypothesize that the static motor control group will improve more on this outcome than the sensor-based movement control group.

To confer clinical benefit beside movement control of the spine, we will also evaluate differences between the offered interventions in terms of therapy adherence, their respective effects on disability, pain intensity, fear avoidance beliefs, and health related quality of life.

## Methods

### Design

**Figure 1:**
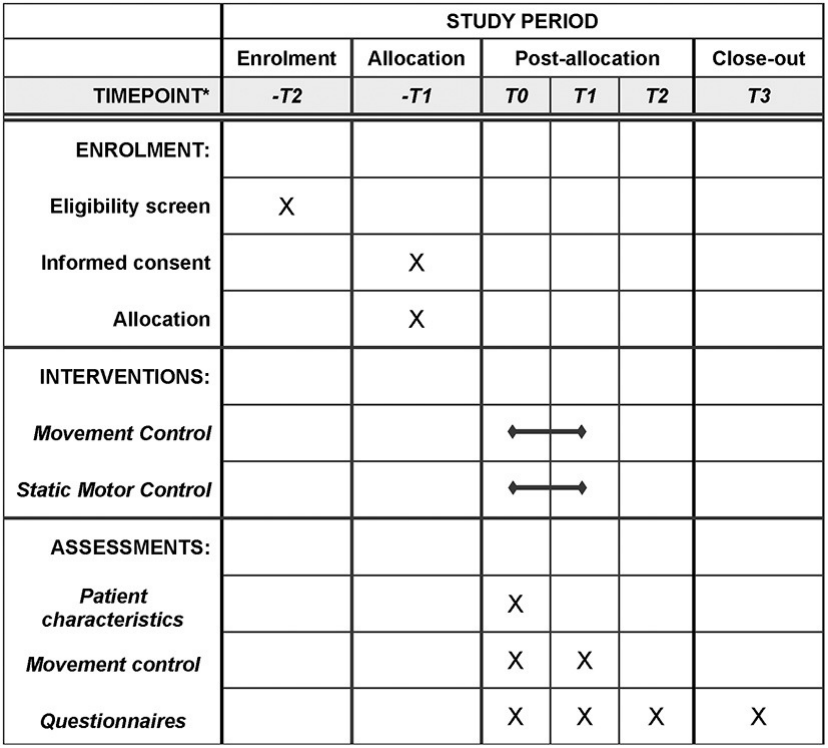
Flowchart for participants of this study. *-T2 two weeks for T0; -T1 one week for T0; T0 week 1; T1 week 10; T2 week 26; T3 week 52.

In this single-center quasi-randomized controlled trial, 60 low back pain patients will be quasi-randomly assigned to either the Sensor-Based Movement Control group (*n* = 30) or the Static Motor Control group (*n* = 30) (Figure 1). Both interventions are nested within a 12-week multidisciplinary rehabilitation programme for low back pain at the Military Rehabilitation Centre “Aardenburg” (MRC), Doorn, The Netherlands. This study protocol was approved by the METC Brabant (protocol number NL76811.028.21). Informed consent will be obtained from all patients prior to entry into the study by one of the investigators (BM, LV, MP). This trial received funding of the Stichting Ziektekostenverzekering Krijgsmacht (SZVK) in the Netherlands. This study design follows the recommendations of SPIRIT 2013 (Supplementary material 1).

### Study setting

Patients will be recruited from both the inpatient and outpatient population of the MRC. Approximately 100 low back pain patients are treated in the Centre each year (21). With an inclusion rate of 80% and a dropout rate of 20%, inclusion can be completed in ~1 year and the final follow-up can be completed ~2 years after the start of the study. Enrolment started on 17 May 2021 and is ongoing. Data collection is in progress.

### Patient and public involvement

There has been no patient and public involvement as co-producers of this study.

### Randomization, blinding and treatment allocation

This study is quasi-randomized and non-concealed. Patients will be screened in the first week of the multidisciplinary rehabilitation programme (2 weeks prior to baseline data collection). Enrolment and allocation will be 1 week prior to baseline data collection after consent of the patient. Patients will be enrolled and allocated to each intervention by one of the investigators (BM, LV, MP) based on the starting date of the multidisciplinary rehabilitation programme. The research team has no influence on the starting date of each patient. If two patients start on the same date, allocation order will be alphabetically (based on the patients last name). The first five patients were allocated to the static motor control group, followed by five patients in the sensor-based movement control group and so forth until 30 patients have completed each intervention. This random allocation sequence was chosen by the investigators to keep group therapy planning feasible. Patients and therapists will not be blinded as this is practically impossible, however they will not be informed about the hypotheses of the study. The primary study outcome (spinal movement tracking error) will be calculated using a computer algorithm (custom made in D-flow, Motek, Amsterdam, The Netherlands) that will work independent of treatment allocation. Investigators involved in the statistical analysis will not be blinded to group. The clinical movement control battery tests will be recorded on video and scored by two examiners that are blinded to time (before/after intervention) and allocation.

### Participants

The in- and exclusion criteria of this study are presented in Table 1. Patients will be screened by a physician and a manual therapist at the MRC. Based on history, physical examination and evaluation of at least one medical image obtained in the past 12 months (X-ray, CT, MRI), serious pathology of the spine will be excluded. A high Body Mass Index (BMI) could hamper the planned sensor-based movement control intervention as a result of movement artifacts (22); therefore, patients are excluded if the BMI is higher than 35 (kg/m^2^). To avoid the risk of electromagnetic interference with the inertial sensors, patients with implanted electronic devices of any kind are excluded from this study (23).

**Table 1:** In- and exclusion criteria.

**Inclusion criteria**
Between 20 and 60 years of age
Experienced low back pain on a daily basis over the last 3 months, with or without accompanying leg pain above the knee
**Exclusion criteria**
Any condition (other than chronic low back pain) that might interfere with motor control of the spine
A recent (< 5 years) surgical intervention of the spinal column or a spinal fusion.
Proven serious pathology of the spine and related structures, infections, recent fractures
Psychiatric disorders
Signs of neurological compression; loss of sensory or motor functions in the legs and/or pelvis and/or radiating pain in the lower leg and/or foot
The use of drugs that influence the reaction time
A body mass index of 35 (kg/m^2^) or more
Implanted electronic devices of any kind

### Sample size

The main objective of this study is to assess if a Group x Time interaction effect exists for movement control of the spine, i.e., if movement control of the spine changes differently between groups over the course of the intervention. To the best of our knowledge, currently no studies have been performed in which spinal movement control, as defined in the current paper, is both trained and assessed before and after training, which complicates the estimation of an expected effect size. For the study outcome to be clinically meaningful we have set the goal to detect or reject an arbitrary effect size of 0.25 (24). In other words, if the effect size would be below 0.25, we would consider this result to be too small to be of interest. Differences in movement control of the spine in low back pain patients and healthy controls in terms of tracking error of a spinal movement controlled tracking task were reported by Willigenburg et al. (12). The tracking error in healthy controls was 0.332 degrees (SD 0.103) and in low back pain patients 0.422 degrees (SD 0.634), which is a large effect size (>0.8). No data about the expected effect of a sensor-based movement control intervention on these outcomes are available. However, a recent study from Matheve et al. demonstrated that low back pain patients can alter their movement behavior using a sensor-based intervention during a single session (14). Hence, we expect that low back pain patients will be able to perform equally well on movement control tasks by the end of the intervention as healthy controls without an intervention. If the static motor control group reaches 75% of the effect of the sensor-based movement control group, corresponding to an effect size of 0.25 (considering the effect of the intervention is equally large as the standard deviation of the effect), a total sample size of 54 (27 per group) would suffice to demonstrate a Group x Time interaction effect at a power of 95%. In case of a drop-out an additional patient will be recruited (with a maximum of 10 patients).

### Interventions

The Sensor-Based Movement Control and Static Motor Control intervention will be offered over a course of 8 weeks (weeks 2–9 of the study), each week consisting of two supervised therapy sessions of 20–30 min and four non-supervised home exercises of 5–10 min resulting in nearly one training session every day of the week for 8 weeks.

**Figure 2:**
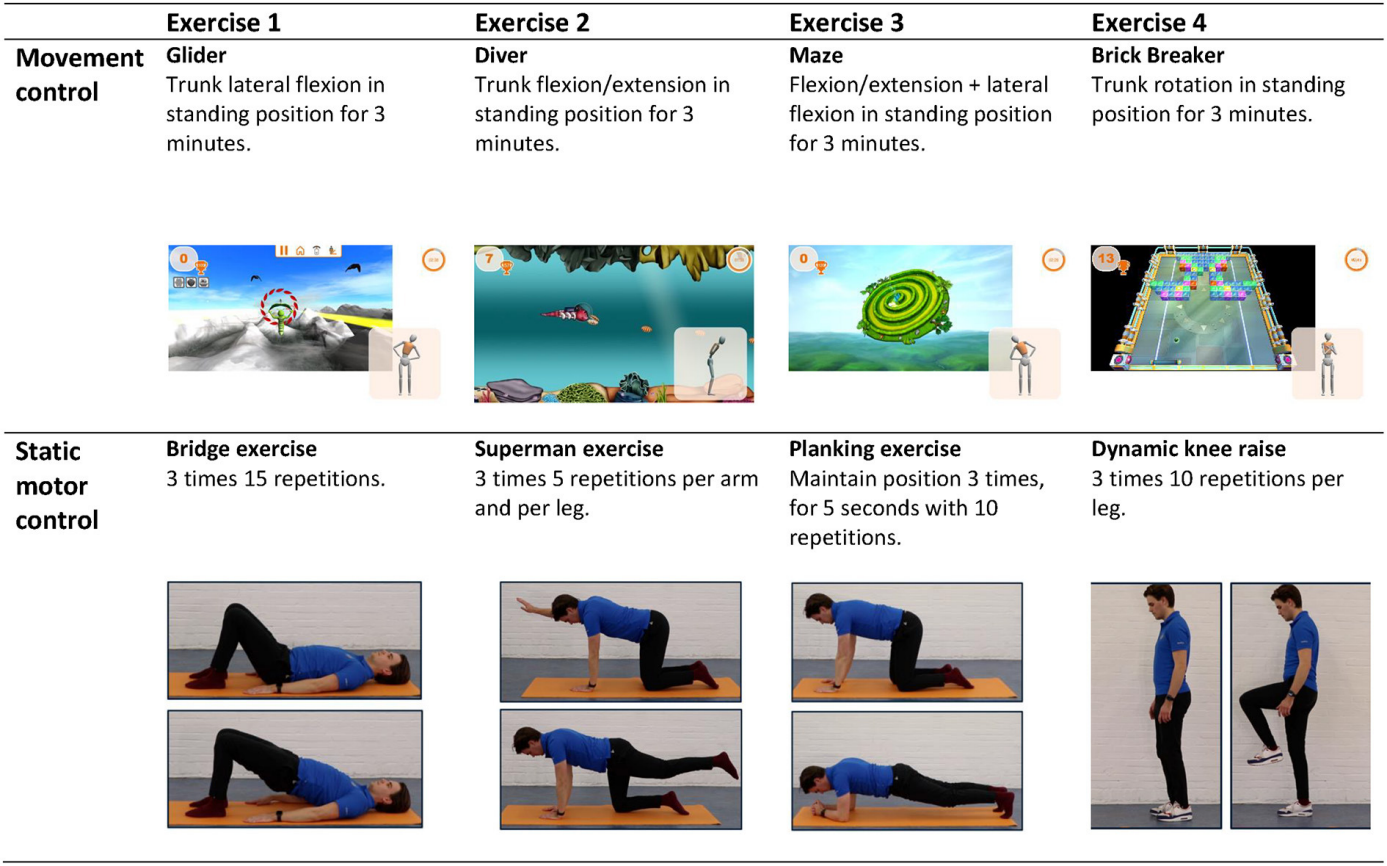
Supervised exercises offered halfway through the intervention (week 4). The exercises of the sensor-based movement control group are displayed in the top row, the exercises of the static motor control group in the bottom row.

The supervised sessions are provided by five experienced (4–10 years) physio- and occupational therapists from the MRC that are trained to provide both the intended interventions. The therapists can also provide the regular therapies in the multidisciplinary rehabilitation programme of a patient. Three training moments are given to all therapists at the same time by the investigators to understand the content of the interventions and how to offer this to the patients during 8 weeks. The quality will be assured by several evaluation meetings with the investigators and investigators will check the content of the sessions by occasionally being present at the supervised sessions throughout the study. The first four sessions will be individual, i.e., one patient supervised by one therapist. In these sessions, the capacity of the patient will be determined by the therapists and the patient will get acquainted with the basics of the training. The final 12 sessions will be in groups, with a maximum of three patients in the same intervention per session, supervised by one therapist. The patients will not have the same therapist throughout the programme to keep the rehabilitation planning feasible. The therapists will monitor the progress and challenge the patient if needed during all sessions. There is a standard protocol for exercises throughout the sessions, for both the sensor-based movement control and static motor control group. This protocol was composed by two experienced physiotherapists/human movement scientists and are based on literature (6). Therapists are allowed to modify the standard exercises to match the difficulty level to the capacity of the patient. The standard exercises can also be deviated from to the need of the individual patient as long as it is within the scope of the assigned intervention. There is no standardized approach for modifications in progression, however all modifications will be registered. An example of the protocol for week four is presented in Figure 2. Attendance of the supervised sessions will be registered.

The home exercises consist of four exercises per week, that will be performed on non-therapy days. The non-supervised home exercises are listed in a workbook with text and pictures describing each exercise. In addition, a QR-code is added to each exercise linking to a video-instruction. These video-instructions will be available “unlisted” on YouTube. The therapists will encourage patients in their own manner to do their home exercises. Adherence to the home exercises will be measured by a questionnaire after the intervention and at follow-up at 26 and 52 weeks.

The full protocol of supervised sessions and home exercises is provided in Supplementary materials 2, 3.

#### Static motor control intervention

The supervised therapy sessions and home exercises of the standard static motor control intervention will consist of exercises in which patients will be instructed to contract their m. Transversus Abdominis during a variety of postures and body movements while keeping their spine in neutral position, i.e., trying to make as little spinal movements as possible. The exercises will be offered with increasing intensity, difficulty, and complexity per week by using a variety of postures, movements, and exercise equipment such as a balance board or foam pad to stand on. Modifications in progression of the patient will be registered by the treating therapist. The home exercises will be covered during the supervised sessions to adjust the load level of these exercises to the capacity of the patient if necessary.

#### Sensor-based movement control intervention

For the supervised therapy sessions of the Sensor-Based Movement Control intervention, Valedo^®^ Motion 2.0 (Hocoma) will be used. Valedo Motion is a medical device on which a patient can play games controlled with spinal movements. Spinal movements are tracked using three small inertial measurement units (IMUs), placed on the pelvis (S1), thorax (sternum) and thoracolumbar (L1) area of the spine. The orientation of these sensors is streamed to a laptop and used in real-time to control several games. These games are displayed on a laptop and invite the player to make controlled (in terms of movement direction, speed, and amplitude) movements of the spine in various postures (e.g., standing, sitting or on hands and knees). During the first few sessions, the workflow of the hardware and software will be demonstrated and explained by the therapist. It is expected that the patient can perform the set-up independently (under supervision of the therapist) after these two sessions. During each session, patients will play four different games. Before each session, the patients spinal range of motion around the three anatomical axes will be determined using the software. The standard protocol for games throughout the sessions are pre-set by the investigators and will be offered with increasing intensity, difficulty, and complexity. Modifications in progression of the patient will be registered by the treating therapist. The home exercises resemble the movements and postures of the Valedo games of that week. These exercises will also be adjusted to the capacity and need of the patient by the therapists, for example by changing the game duration or difficulty level of the game or by using exercise equipment.

### Multidisciplinary rehabilitation programme

All patients of the study will be enrolled in a multidisciplinary rehabilitation programme. This is a standard care programme at the MRC for patients with chronic low back pain which has a focus on increasing the activity and physical level, education about back pain, healthy lifestyle and awareness of the body and physical limits. The programme follows a protocol in which the number, duration and content of the therapies is fixed. During this 12-week programme, they will receive multiple therapy sessions for 3 days a week. The programme mainly consists of physiotherapy (19 individual sessions of 30 min), occupational therapy (19 individual sessions of 30 min), sports therapy (20 group sessions of 60 min consisting of fitness, swimming and game sports) and 4 group sessions of body awareness. In the *first* week, pain-education is given by a psychologist and social worker and, if necessary, further individual guidance is provided once a week. The therapists of these disciplines are discouraged, but not prohibited, to focus their interventions on static motor control or spinal movement control exercises and they will not have access to Valedo^®^ Motion during these therapy sessions. Moreover, they are request not to compensate for the given intervention (e.g., providing more dynamic exercises for patients in the static movement control group). The therapists will not be restricted in their therapy programme in any other way. It will not be registered to what extent static motor control or movement control exercises are provided during these therapy sessions.

### Data collection and outcome measures

Patients will be tested at two instances, once before (T0: week 1) and once after the intervention (T1: week 10). In addition, we will contact them by email at 6- and 12-months follow-up (T2: week 26 & T3: week 52). Per follow-up moment, patients receive a maximum of 2 emails and 1 letter, to enhance completion of the follow-up. Table 2 highlights the measures collected at each point in time. At the start of the intervention study, patients' characteristics will be recorded to enable comparison of baseline characteristics of both groups.

**Table 2:** Overview of outcome measurements in this study.

	**Intervention**		**Follow-up**	
	**T0**	**T1**	**T2**	**T3**
**Patient characteristics** Age, height, weight, BMI, gender, duration of complaints	X			
**Questionnaires** RAND-36, FABQ, NRS, ODQ, RMDQ	X	X	X	X
**Movement control assessment** Tracking tasks, clinical spinal movement control tasks, repetitive motion tasks and gait trials	X	X		
**Therapy adherence** EARS		X		

T0: week 1; T1: week 10; T2: week 26; T3: week 52.

#### Primary outcome

**Figure 3:**
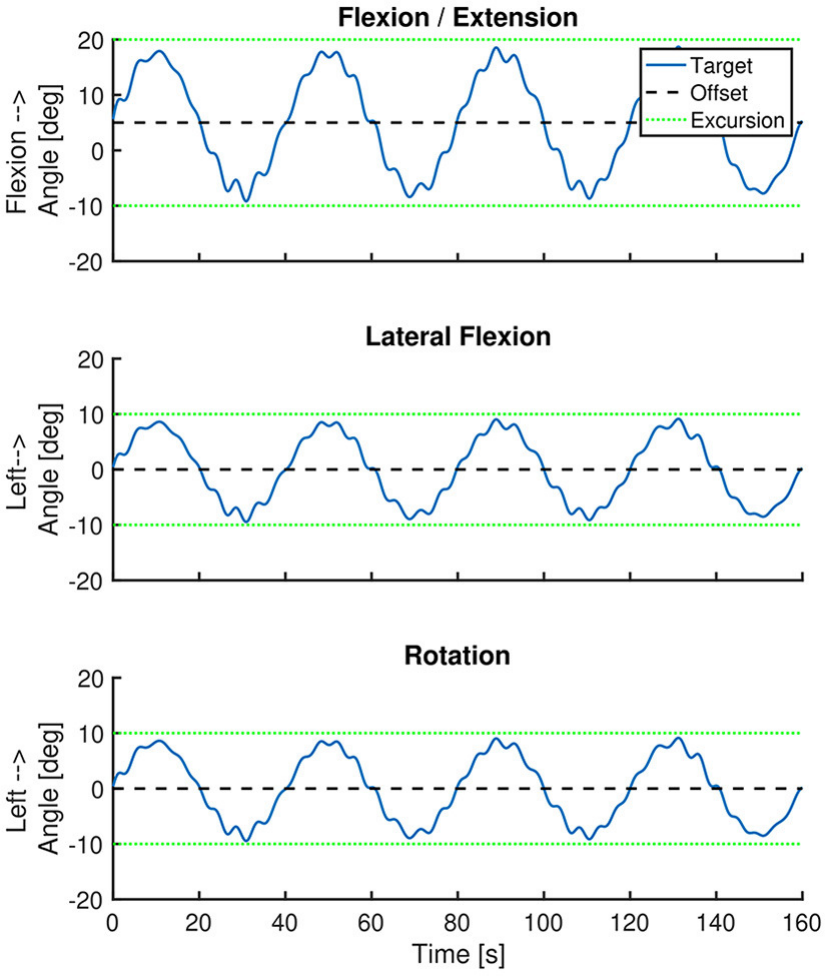
Position of the tracking target over time. The blue line represents the position of the tracking target over time. The black dashed line represents the offset of the target trajectory. The green dotted lines represent the movement excursion during each task. The imposed movement excursion during the lateral flexion and rotation task was symmetrical. During the flexion/extension task the movement excursion was larger in the flexion direction (i.e., 20 degrees) than in extension direction (i.e., 10 degrees).

Our primary outcome measure is movement control of the spine. This will be quantified using three spinal movement controlled tracking tasks and a clinical movement control test battery. The movement controlled tracking tasks are based on the tracking task used in the study of Willigenburg (12). The tracking tasks used in this study consist of one flexion-extension, one lateral flexion and one rotation task, and will be performed at T0 and T1 with 3 Valedo Motion inertial measurement units (IMUs) attached at the right thigh, pelvis (at S1 level) and at the sternum level. During these tasks, patients will be instructed to move their spine in order to keep its real-time representation (on a laptop screen located in approximately one meter in front of them at eye level) within a moving target. Patients are in a seated position. The patient's pelvis will be fixated with a frame, which will be used to guarantee that the spinal angle is changed without any hip motion. Each trial will last 2 min and 40 s, with the first 40 s being for learning the task, and the following 2 min for the actual measurement. During the flexion/extension task, the vertical position of the target on the screen will vary between values that correspond to 20 degrees trunk flexion and 10 degrees trunk extension. During the lateral flexion and rotation task, the horizontal position of the target on the screen will vary between values that correspond to 10 degrees left and 10 degrees right lateral flexion or rotation. In each tracking task, the target will follow a multi-sine with a main frequency of 0.025 Hz (one cycle each 40 s). All these movement excursions are within the maximum range of the tracking task that was used by Willigenburg (12). Formula 1 and Table 3 describe the movement profile of the tracking target in each task, illustrated in Figure 3. The reliability or minimal detectable changes of this movement control measurement is unknown.

**Table 3:** Used offset and ROM for each movement plane and characteristics of the multi-sine-wave.

Movement plane	**Off**set	**ROM**	Excursion
Sagittal (flexion)	5	30	−10/20
Frontal (lateral flexion)	0	20	−10/10
Transversal (rotation)	0	20	−10/10
Sine no.	***Ampl***itude (%)	***f***requency (Hz)	**φ**: Phase (rad)
‘Main' Sine #1	80	0.025	0.00
Sine #2	10	0.215	0.22
Sine #3	6	0.185	0.14
Sine #4	4	0.250	0.84

Bold text in the table corresponds to bold text in Formula 1. ROM: Range of Motion.

***Formula 1: Used offset and ROM for each movement direction and*** ***characteristics of the multi-sine-wave*. ***Bold text corresponds to bold text in* *Table 3**. t* = *time, starting at the beginning of the tracking task*.


$$\begin{array}{l} Target_{plane}(t)=Off_{plane}+\frac{ROM_{plane}}{2}\times \sum_{i_{Sine}=1}^{4}Ampl\left(i_{Sine}\right)\\ \;\times sin\left(2\pi f\left(i_{Sine}\right)t+\varphi \left(i_{Sine}\right)\right) \end{array}$$

The tracking error (average absolute deviation from imposed trunk angle) around the imposed movement axis of the three tasks will be reported as average tracking error (in degrees).

The clinical movement control test battery of the lower back will be the tests of Luomajoki et al., (19, 20). This test battery consists of six active movement control tests in which the patient performs each movement once. The test will be recorded on video from the front or side (depending on the movement) using a video-recorder and two blinded experienced (>10 years) physiotherapists will rate the performance of the tests from the recordings. These therapists will be instructed by the investigators how to score this assessment prior to the ratings. The total score can range from 0 to 6, indicating the number of tests with clear movement dysfunction. The final score of the test battery will be calculated as a mean of the two raters.

#### Secondary outcomes

Spinal movement will also be measured in other movement tasks as secondary outcomes.

– Gait trials: Cycle-to-cycle variability of spinal rotations (measured in degrees) will be measured during gait on a treadmill. The tasks consist of walking for 5 min at three different walking speeds, with a small pause for IMUs calibration in between: one at comfortable walking speed, one at 6 km/h (“fast walking”) and one at 2 km/h (“slow walking”) (25).
– Repetitive bending task: Cycle-to-cycle variability of spinal flexion (measured in degrees) will be measured during a repetitive bending task. Patients will touch the sides of a box (2 cm x 30 cm x 30 cm) that is in front of them, at their tibial tuberosity height, for 40 times. Prior to the task, a video of the task executed at 0.92 Hz is shown to give patients a visual demonstration of the expected task movement and frequency.
– Repetitive rotation task: Cycle-to-cycle variability of spinal rotation will be measured during a repetitive standing rotation task. Patients will touch two lateral targets with their contralateral hands, alternating them for 80 times (40 times each). Targets' positions are at the patients' homolateral arm distance, at their shoulders' height, and rotated 45° relative to their anteroposterior axis. A video of the task executed at 0.62 Hz is shown to give patients a visual idea of the task's expected movement and frequency.
– Self-developed movement control test of the lower back. During this test, patients will perform 4 movements consisting of 3 continuous repetitions of a pelvic tilt, flexion/extension, lateral flexion and rotation of the spine. First, all 4 movements will be performed seated and next, these same 4 movements will be performed in standing position resulting in 8 tests. Each test will be scored in “correct” (two points): low back or pelvic movement is performed fluently and isolated (thoracic movement in absence of pelvic movement or vice versa); “partial correct” (one point): the movement is performed not fluently *or* insufficiently isolated; or “not correct” (zero points): the movement is not performed fluently nor isolated. A higher score represents a better movement control of the lumbar spine. The test will be recorded on video and rated in the same manner as the movement control test battery of Luomajoki. This test was developed because in the test battery by Luomajoki et al. (19, 20) no movement of the spine is requested during most tests. In fact, in five out of the six imposed movements, the subjects are instructed explicitly to not move the lumbar spine.

We have no specific hypotheses regarding the aforementioned movement variability outcomes. These outcomes were primarily assessed as part of a case control study (see pre-registration https://osf.io/3dr58).

The tracking tasks, gait trials and repetitive motion tasks will be performed in quasi-random order at baseline and post-intervention measurements.

In addition to the movement control outcomes, patient reported outcome questionnaires will be assessed at baseline, post-intervention and at follow-up. The patient reported outcomes measured in this study will be: Exercise Adherence Rating Scale (EARS) (26), Dutch version of the Oswestry Disability Index (ODI) 2.1a (27), Dutch version of the Roland Morris Disability Questionnaire (RMDQ) (28, 29), three scores of Numeric Rating Scale (NRS) for Pain; the average and maximum pain intensity over the past 7 days and current pain intensity (29), Dutch version of Fear Avoidance Beliefs Questionnaire (FABQ) (30), Four scales of the Dutch version of the RAND-36 (Physical functioning, mental health, general health and pain) (31).

### Data management

Patients will receive a unique three-digit number that will be used on all forms (except the informed consent form) used in this study. Only the principal investigators will have access to the key of this code list. The informed consent and patient related forms will be stored separately from the other forms and will be stored for 15 years. Video-recordings of the clinical movement control test battery will be stored locally at the MRC. Only the principal investigators will have access to these recordings. The recordings will be scored by two independent physical therapists, under supervision of a principal investigator.

### Statistical analysis

To evaluate if movement control of the spine changes differently between groups over the course of the treatment, repeated measures ANOVAs will be used to evaluate if a significant Group x Time interaction effect exists for the tracking error during each tracking task separately, the average tracking error of the tracking tasks and the total score of the clinical movement control test battery. The main effects of Group and Time will also be assessed using the same ANOVA. In addition to the total score of the clinical movement control test battery by Luomajoki et al. (19, 20) the performance on the individual tests that comprise the test battery will also be reported per group at T0 and T1.

Secondary study parameters will be assessed in the same manner as described above without correction for multiple testing, because these analyses are of an exploratory nature, and we want to limit the probability of type 2 errors. For the questionnaires that will be filled out on more than two occasions, we will perform *post-hoc* independent t-tests, with LSD correction, comparing results between groups at each point in time. Patient characteristics and all questionnaires filled out during the first testing day will be compared between groups using independent sample *t*-tests without correction for multiple testing to evaluate if differences existed at baseline. Statistical analyses in this study will not be adjusted for baseline differences between groups, as recommended by de Boer et al. (32). Independent of normality, parametric statistics will be used in this study and the alpha level will be set at 0.05 (33).

Data from patients who attended less than 10 sessions are not included in the statistical analysis. In addition, the data is also not included if patients have dropped out of the study before T1.

Missing data will be handled by using complete case analysis with all repeated measures ANOVAs between T0 and T1 and independent *t*-tests between T0, T1 and T2 and between To, T1, T2 and T3.

All statistical analyses will be performed using R version 4.1.1.

### Adverse events

Adverse events are defined as any undesirable experience (harmful, objectionable, or unpleasant) occurring to a patient during the study, whether or not considered related to the testing procedures or the experimental intervention. In the study information letter patients are instructed to contact the investigators in case of an adverse event. All adverse events reported spontaneously by the patient or observed by the research team will be recorded. A serious adverse event is any untoward medical occurrence or effect that results in death; is life threatening (at the time of the event); requires hospitalization or prolongation of existing inpatients' hospitalization; results in persistent or significant disability or incapacity; any other important medical event. The investigator will report all SAEs to the sponsor without undue delay after obtaining knowledge of the events.

### Data monitoring

The study will be terminated prematurely if decided so by: the board of physiatrists from the MRC or the board of the MRC. There is no data monitoring committee or independent audit for this study.

## Discussion

In this study, the effect of a sensor-based spinal movement control intervention on the movement control of the spine in low back pain patients over the course of a multidisciplinary rehabilitation programme will be compared to the effect of conventional static motor control exercises. In addition, we aim to evaluate the effect of the intervention on disability, pain intensity, fear avoidance beliefs and health related quality of life. Finally, therapy adherence will be compared between the interventions. Sensor-based exergames are a relatively new tool to train spinal movement control using meaningful and engaging feedback. To our acknowledge, this is the first study which evaluates if sensor-based exergames training influences movement control of the spine in low back pain patients to a greater extent than static motor control training.

Currently, there is no gold standard to assess movement control of the spine. Therefore, we will analyse our main outcome with three different assessments: [1] sensor-based tracking tasks on a laptop, based on a tracking task from Willigenburg et al. (12), [2] the clinical movement control test battery of Luomajoki et al. (19, 20) and [3] a self-developed clinical movement test battery. Because these tests are performed in the same subjects at the same moment, the results of this study could provide us more insight in how to assess movement control of the spine. The reliability and minimal detectable changes of the movement control tracking tasks and the self-developed clinical movement test battery are not available which may bias the outcome of the study.

There are several limitations of this study that need to be addressed. Our study population completely consists of Dutch military personnel. The Dutch military population mostly consist of males, who are relatively young and physically active compared to the civilian population (34). The cause of low back pain in this population is mostly overuse, due to the high workload in the Netherlands Armed Forces (34, 35). For this reason, the generalizability of the results of this study might be compromised. Another limitation, from a clinical perspective, is that the main outcome of this study (spinal movement control) does not correspond to the main focus of most patients, which is reducing pain and/or disability. Although these outcomes will be assessed, the study might be underpowered to demonstrate significant effects on these domains for at least two reasons. First, some patient subgroups may derive a greater benefit from one type of exercise than another (e.g., static motor control vs. sensor based movement control) because of the heterogeneity in the low back pain population. Exploratory analyses can be performed to evaluate if these subgroups appear to exist, but a larger sample is expected to be required to provide more conclusive evidence. Second, because this study is embedded in a multidisciplinary rehabilitation program, an effect of the tested intervention on pain or disability might be hidden by the effect of other components of the program. Finally, for the main outcome of this study, movement control of the spine, the effect of other therapies might reduce the contrast between groups as well. It cannot be excluded that patients in the static motor control group will also receive exercises of the movement control group during other therapies and vice versa. This can be considered a study confounder. However, we hypothesize that it is relatively difficult to improve spinal movement control without the use of sensors. Embedding this study within a multidisciplinary rehabilitation program could be considered a test of this assumption. Each week of the intervention consists of two supervised therapy sessions of 20–30 min and four non-supervised home exercises of 5–10 min. Although therapists will encourage patients to do their home exercises, compliance can influence results in this study. For this reason, self-reported exercise adherence will be measured in both groups.

The results of this study will help to inform clinicians and researchers on the efficacy of movement control training in combination with multidisciplinary rehabilitation programme for patients with low back pain. Also it will enlighten preliminary impacts of the interventions on patient reported outcomes. This could directly affect decision making in clinical practice and culminate in larger trials to assess if pain and/or disability could be reduced by movement control training in (subgroups of) low back pain patients.

## Ethics and dissemination

### Ethical considerations

This study will be performed in accordance with the Declaration of Helsinki. Ethical approval was obtained from the Medisch Ethische Toetsingscommissie (METC) Brabant on 14 Mai 2021 and all procedures will be conducted in accordance with the statement conducting research involving humans. Informed consent will be obtained by the investigators from all potential patients and patients will be aware that participation is voluntary and can withdraw from the study at any time.

### Safety considerations

The tests at the beginning and end of the intervention could result in a transient increase in low back pain. Training with sensors could result in spinal tissue overload as a result of lack of focus on bodily sensations. However, the Military Rehabilitation Center has more than 10 years of experience with providing a similar type of therapy in low back pain patients. Moreover, the complexity, duration and intensity of the exercises will be increased gradually, which would minimize the chance of overloading the spinal structures. Damage to research subjects through injury or death caused by the study is covered by the Ministry of Defense. This applies to the damage that becomes apparent during the study or within 4 years after the end of the study.

### Dissemination

To protect confidentiality, personal information about the patients will be collected, shared and maintained in a database on a secured computer that can only be accessed by principal investigators before, during and after the trial.

Any significant modifications of the study protocol will be communicated to the METC, trial funder (SZVK), Open Science Framework Registries and the trial sponsor (MRC). The investigators will communicate trial results to the patients, trail sponsor, METC and funder within 1 year after the end of the study. The study results shall be presented at symposia, conferences and to publish in journals and theses without publication obligations from the sponsor.

## Ethics statement

The studies involving human participants were reviewed and approved by Medisch Ethische ToetsingsCommissie (METC) Brabant. The patients/participants provided their written informed consent to participate in this study. Written informed consent was obtained from the individual(s) for the publication of any potentially identifiable images or data included in this article.

## Author contributions

BM, LV, SB, JD, and MP contributed to conception and design of the study. BM and LV are the coordinating researchers and responsible for recruitment, randomization, data collection, and writing the first draft of the manuscript. MP supervises the study and also drafts sections of the manuscript. All authors contributed to manuscript revision, read, and approved the submitted version.

## Funding

This study has received funding from Stichting Ziektekostenverzekering Krijgsmacht (SZVK) under the grant agreement number 20-0129. The funder had no role in the design of the study and collection, analysis and interpretation of data and in writing the manuscript.

## Conflict of interest

The authors declare that the research was conducted in the absence of any commercial or financial relationships that could be construed as a potential conflict of interest.

## Publisher's note

All claims expressed in this article are solely those of the authors and do not necessarily represent those of their affiliated organizations, or those of the publisher, the editors and the reviewers. Any product that may be evaluated in this article, or claim that may be made by its manufacturer, is not guaranteed or endorsed by the publisher.
