# Supplementary material for: Sensor-based intervention to enhance movement control of the spine in low back pain: Protocol for a quasi-randomized controlled trial
Source: Front Sports Act Living. 2022 Oct 17;4:1010054. doi: 10.3389/fspor.2022.1010054 (PMC9619097; doi:10.3389/fspor.2022.1010054)

Supervised Exercises Sensor-Based Movement Control intervention (week 1 – 8)

Overview of all training exercises with Valedo Motion Sensors used in this study. A full guideline of 4 exercises per week.

Week 1

Range of Motion

Lateral flexion, flexion extension, rotation, sagittal tilt and lateral tilt

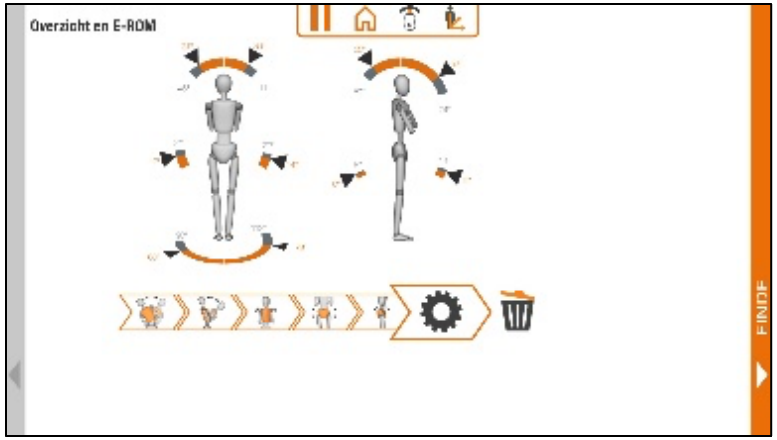

Diver Pelvis Sagittal Tilt

Position: Sitting Minutes: 3 Level: 1

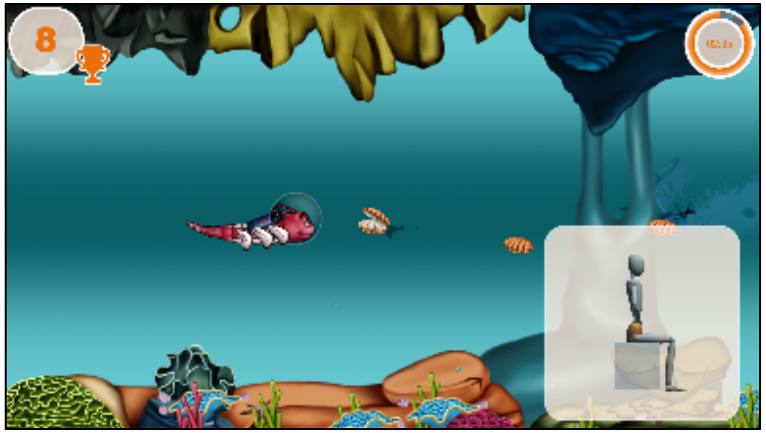

Cave Diver Pelvis Sagittal Tilt

Position: Crawling Minutes: 3 Level: 1

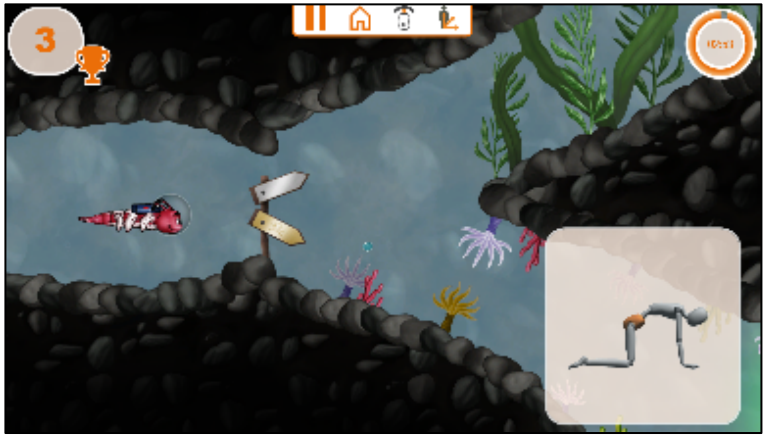

High Flyer Pelvis Sagittal Tilt

Position: Sitting Minutes: 3 Level: 1

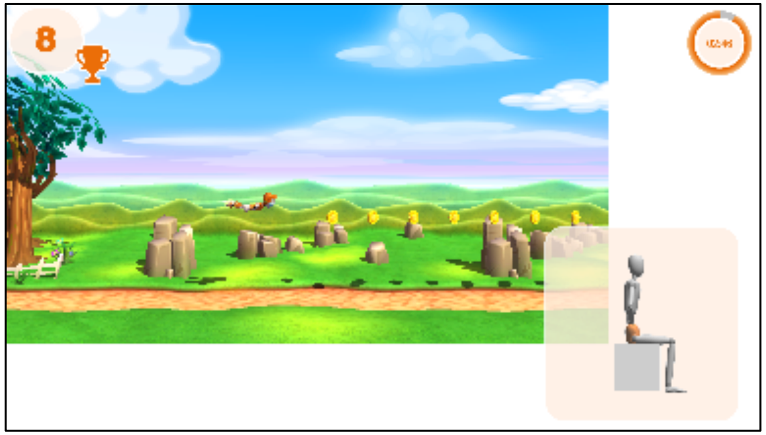

Week 2

Diver Pelvis Sagittal Tilt

Position: Sitting Minutes: 3 Level: 3

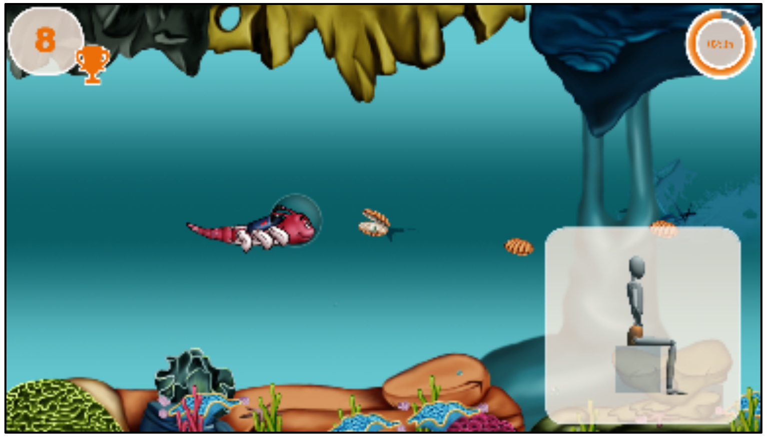

Glider Pelvis Lateral Tilt

Position: Crawling Minutes: 3 Level: 1

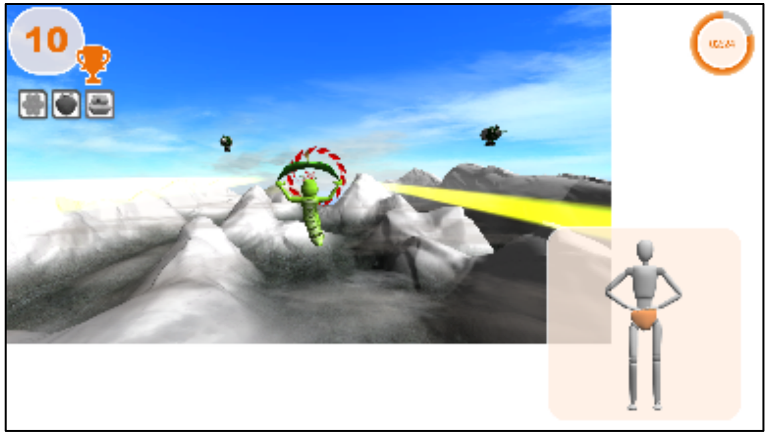

High Flyer Pelvis Sagittal Tilt

Position: Standing Minutes: 3 Level: 1

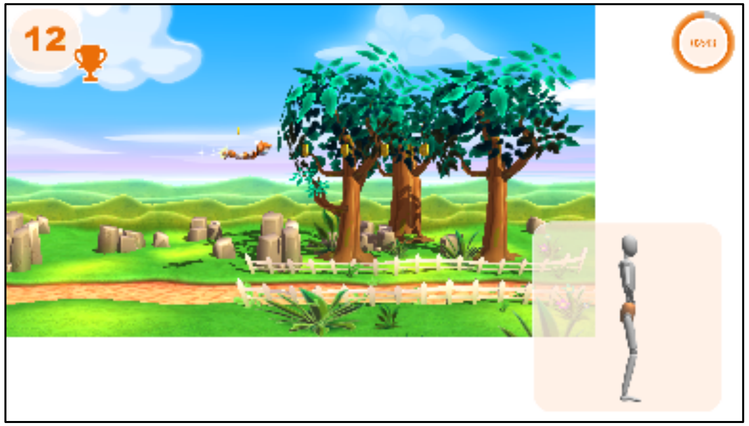

Brick Breaker Trunk Rotation

Position: Sitting Minutes: 3 Level: 1

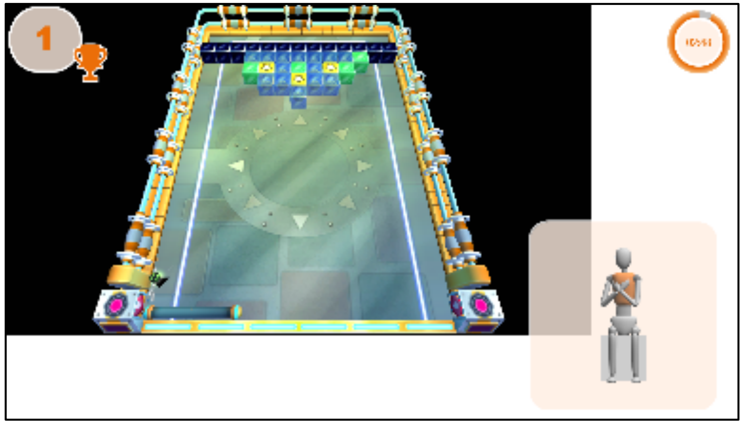

Week 3

Glider Pelvis Lateral Tilt

Position: Crawling Minutes: 3 Level: 1

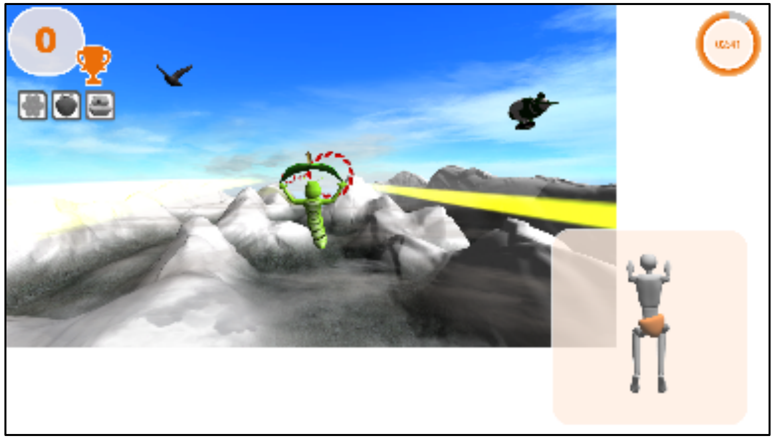

High Flyer Pelvis Sagittal Tilt

Position: Standing Minutes: 3 Level: 2

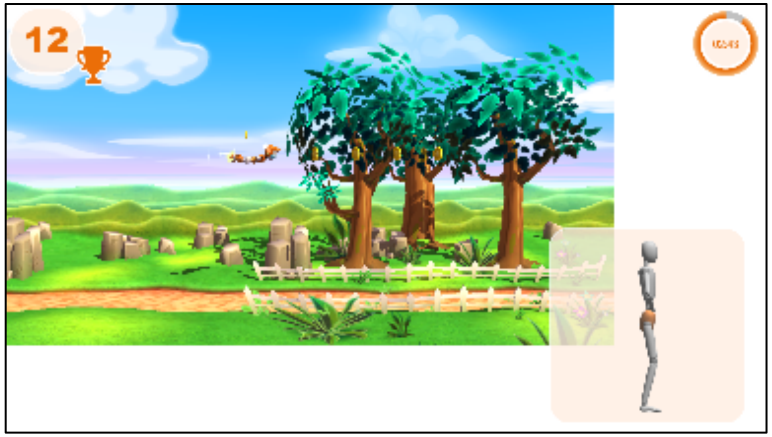

Maze Pelvis Lateral + Sagittal tilt

Position: Standing Minutes: 3 Level: 1

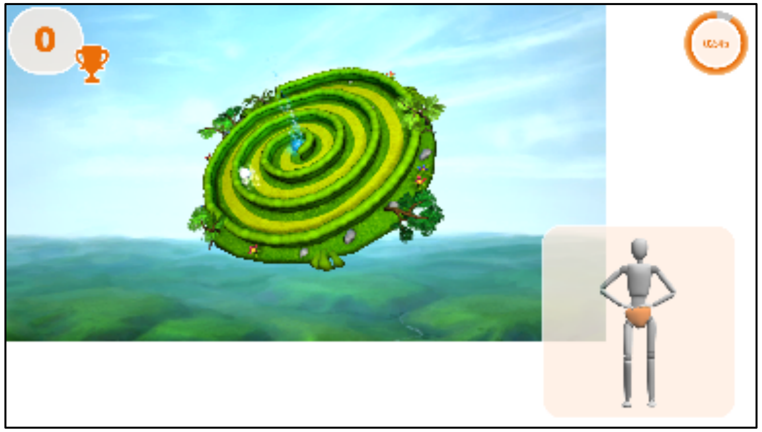

Brick Breaker Trunk rotation

Position: Sitting Minutes: 3 Level: 2

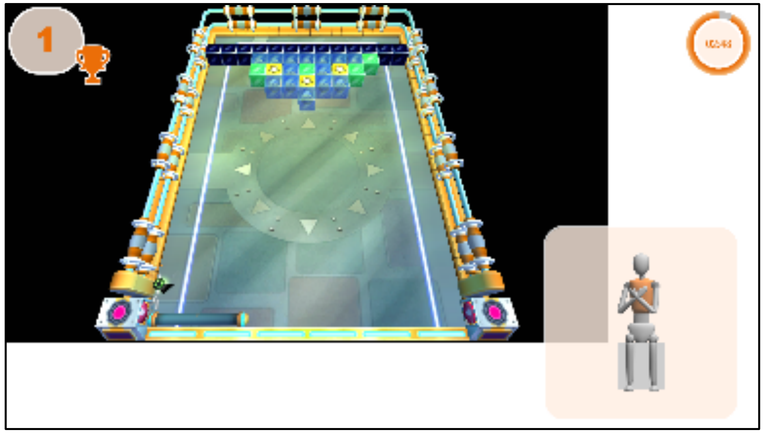

Week 4

Diver Trunk Flexion Extension

Position: Standing   Minutes: 3   Level: 1

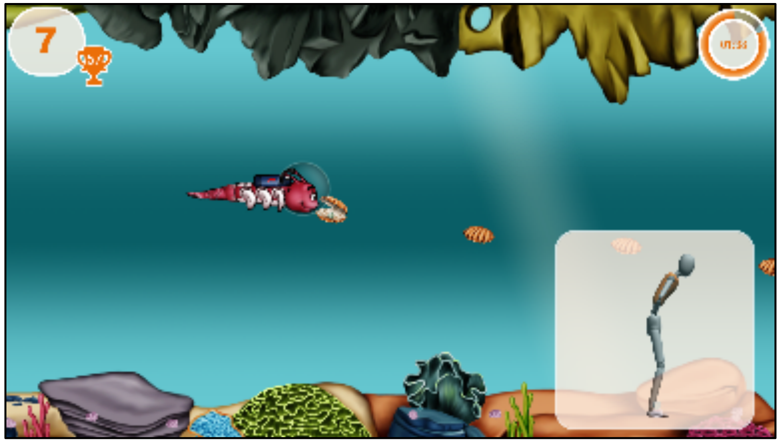

Glider Trunk Lateral Flexion

Position: Standing   Minutes: 3   Level: 1

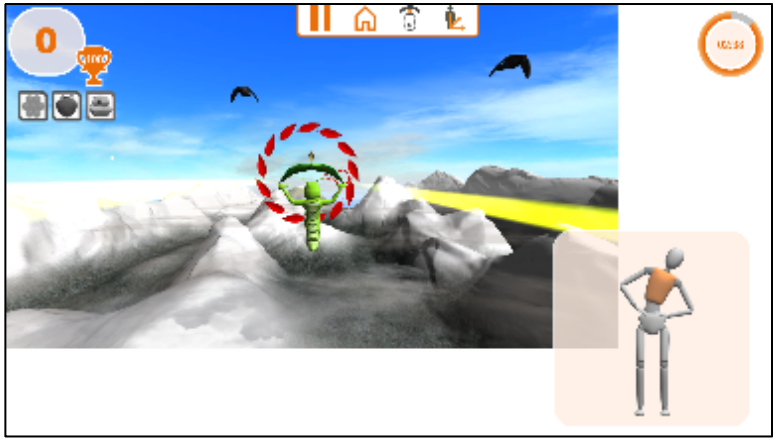

Maze Trunk Flexion Extension + Lateral Flexion

Position: Standing   Minutes: 3   Level: 1

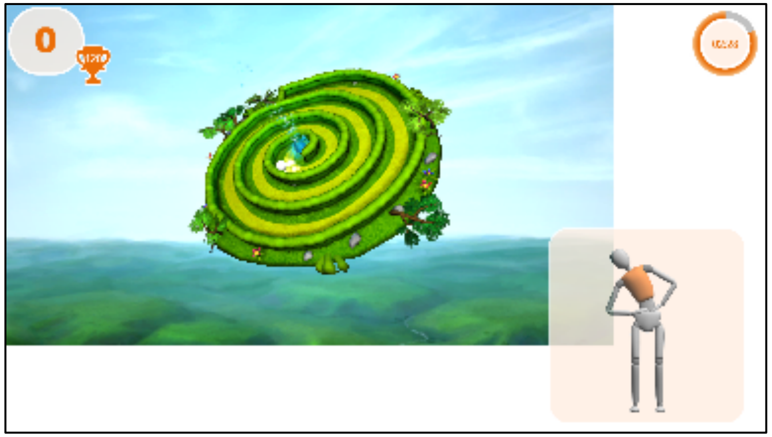

Brick Breaker Trunk Rotation

Position: Standing   Minutes: 3   Level: 2

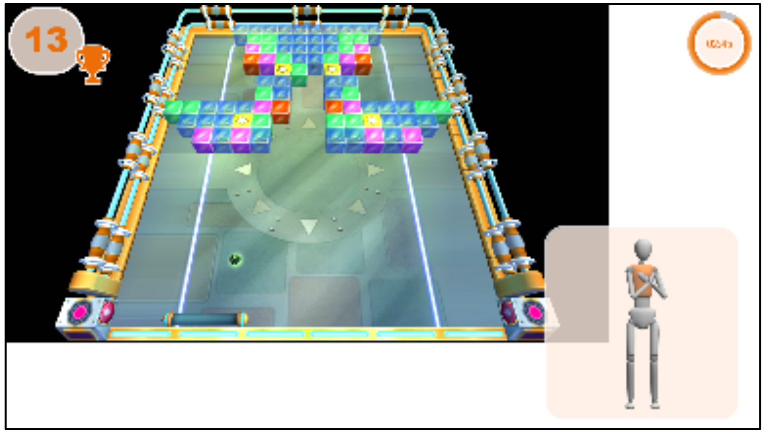

Week 5

Golf Trunk Flexion Extension + Lateral Flexion

Position: Standing Minutes: 3 Level: 1

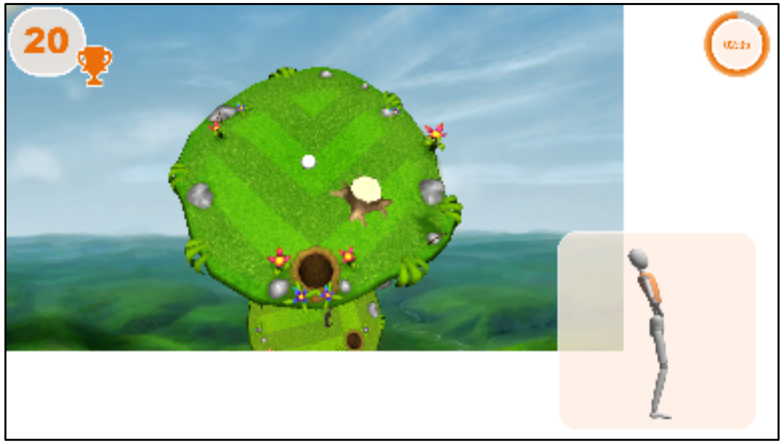

High Flyer Pelvis Sagittal Tilt

Position: Standing Minutes: 3 Level: 2

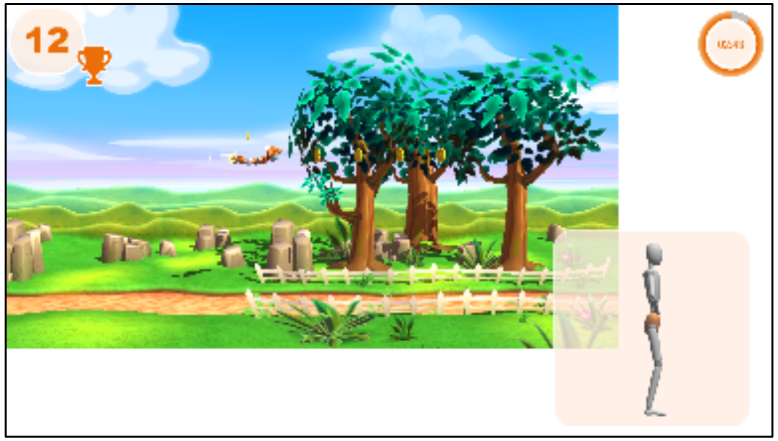

Colors Pelvis Lateral + Sagittal tilt

Position: Crawling Minutes: 3 Level: 1

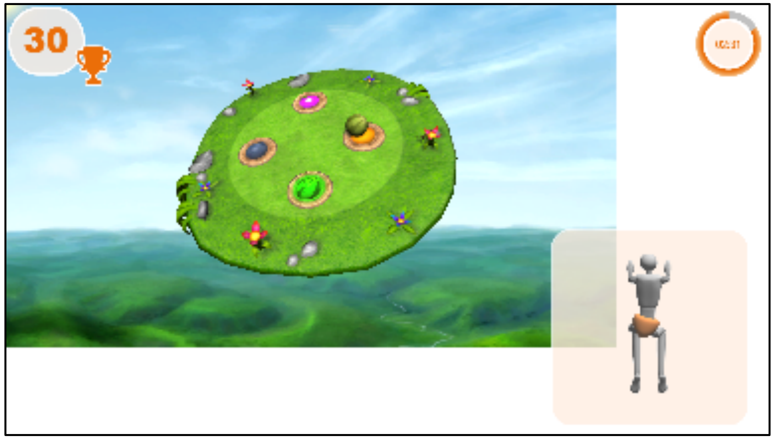

Brick Breaker Trunk Rotation

Position: Standing Minutes: 3 Level: 3

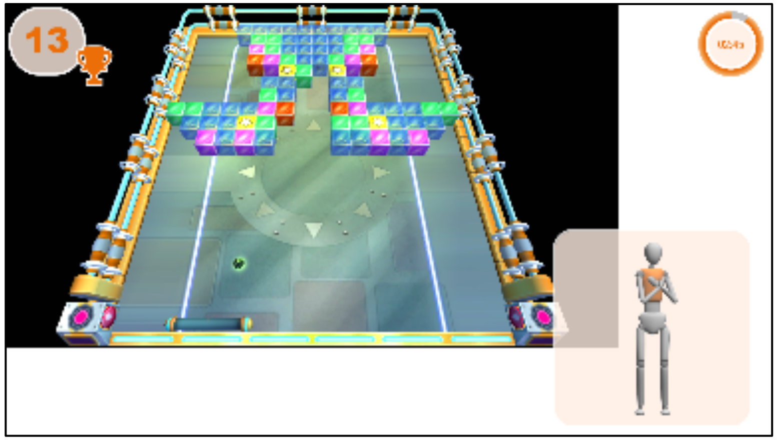

Week 6

Golf Trunk Flexion Extension + Lateral Flexion

Position: Standing Minutes: 3 Level: 3

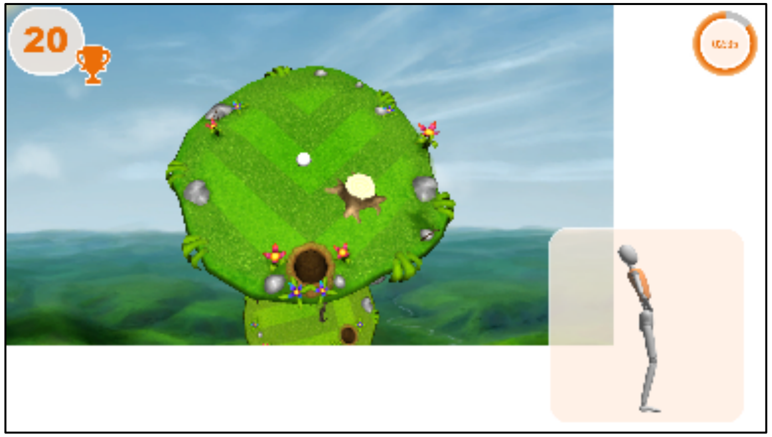

Cave Diver Pelvis Sagittal Tilt

Position: Crawling Minutes: 3 Level: 2

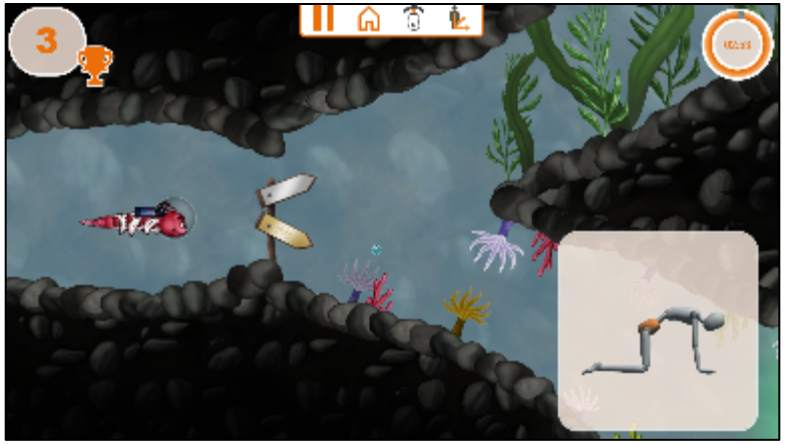

Treasure Island Trunk Flexion Extension + Lateral Flexion

Position: Standing Minutes: 5 Level: 1

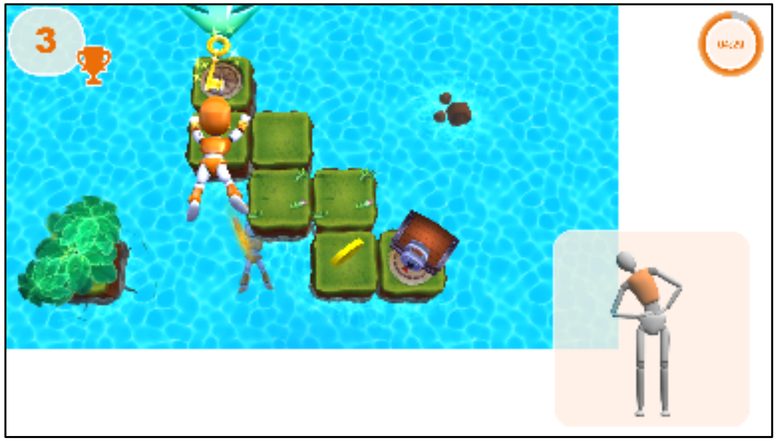

Brick Breaker Trunk Rotation

Position: Standing Minutes: 3 Level: 3

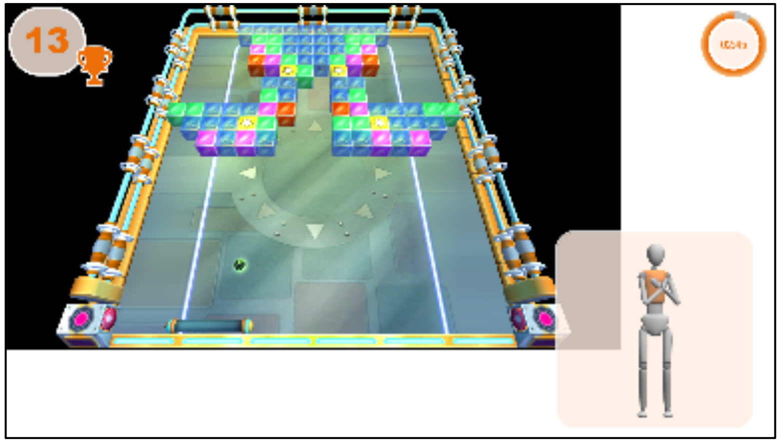

Week 7

High Flyer Trunk Flexion Extension

Position: Standing Minutes: 3 Level: 3

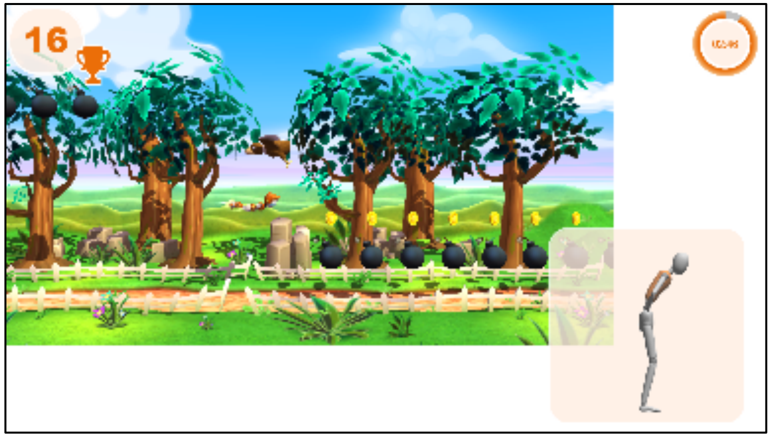

Clock Trunk Flexion Extension + Lateral Flexion

Position: Standing Minutes: 3 Level: 2

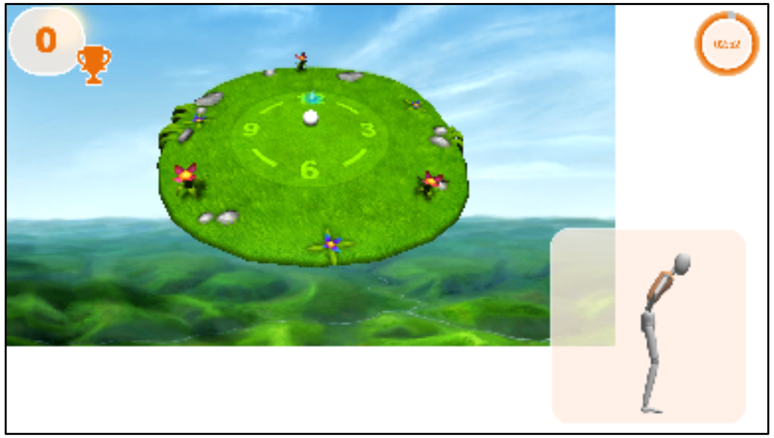

Maze Pelvis Sagittal Tilt

Position: Standing Minutes: 3 Level: 3

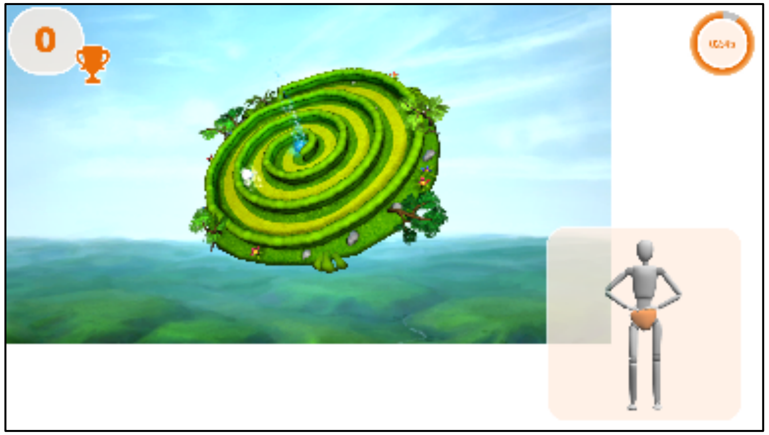

Treasure Island Trunk Flexion Extension + Lateral Flexion

Position: Standing Minutes: 3 Level: 3

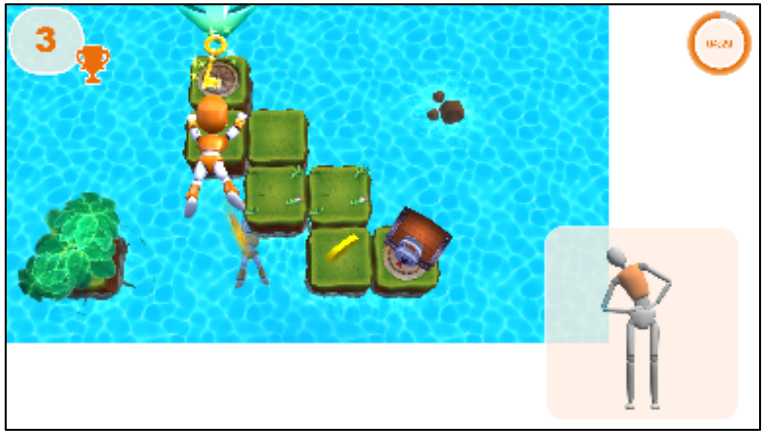

Week 8

Glider Pelvis Lateral Tilt

Position: Standing Minutes: 3 Level: 3

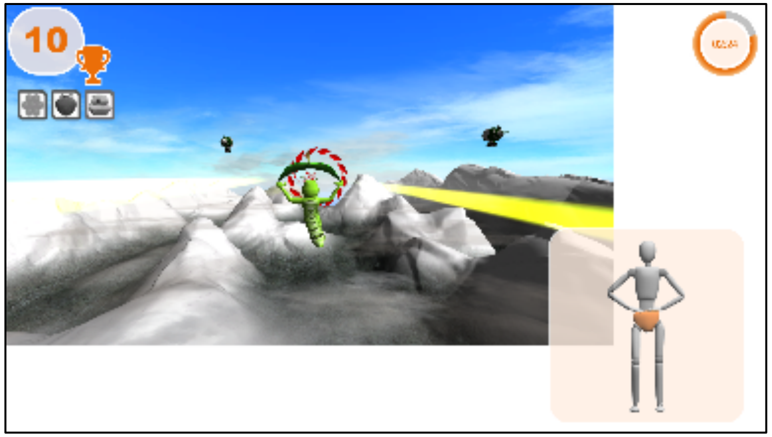

High Flyer Pelvis Sagittal Tilt

Position: Sitting Minutes: 3 Level: 3

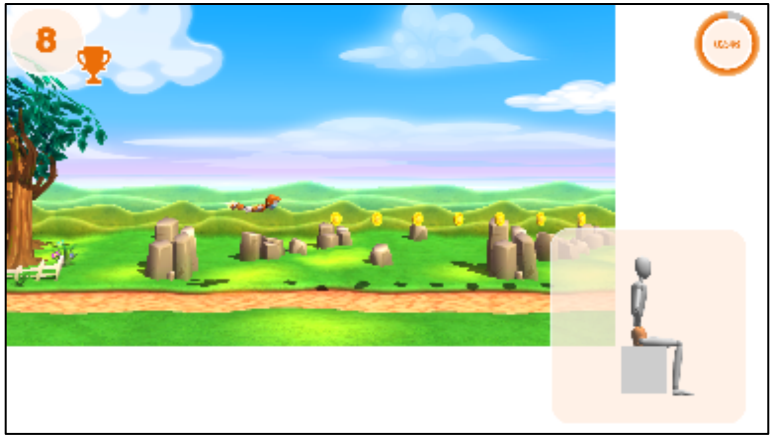

Treasure Island Trunk Flexion Extension + Lateral Flexion

Position: Sitting Minutes: 3 Level: 3

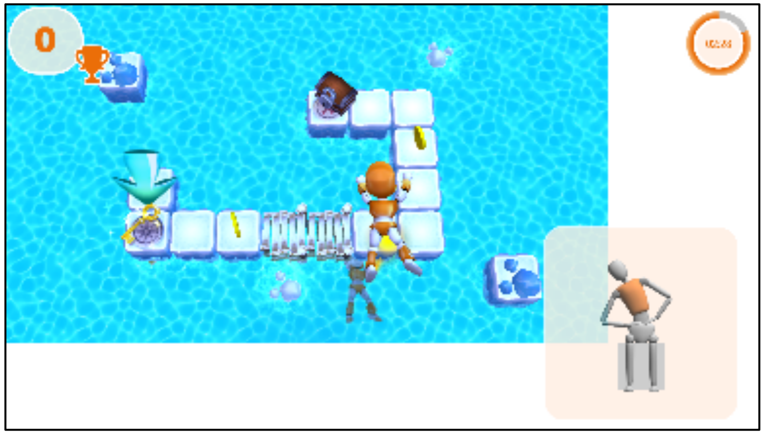

Brick Breaker Trunk Rotation

Position: Standing Minutes: 3 Level: 3

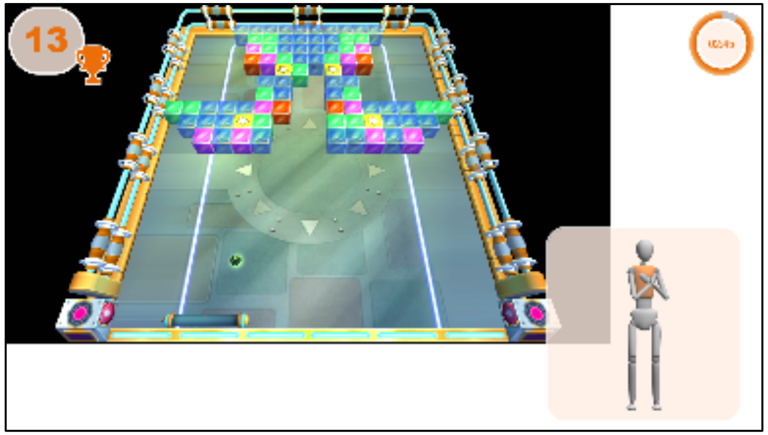

Supplement: Supplementary file 1 [file Data_Sheet_1.pdf]
