# Supplementary material for: Sensor-based intervention to enhance movement control of the spine in low back pain: Protocol for a quasi-randomized controlled trial
Source: Front Sports Act Living. 2022 Oct 17;4:1010054. doi: 10.3389/fspor.2022.1010054 (PMC9619097; doi:10.3389/fspor.2022.1010054)

Home exercises Sensor-Based Movement Control intervention (week 1-8)

Overview of all movement control home training exercises used in this study. A full guideline of 4 exercises per week.

Week 1

Pelvic tilt
15 repetitions.

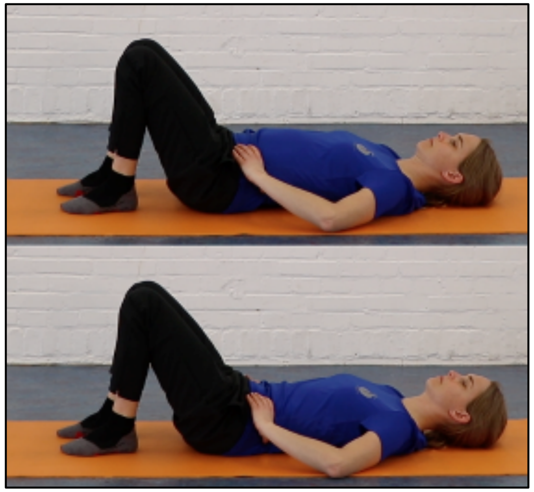

Pelvic tilt in crawling position
15 repetitions.

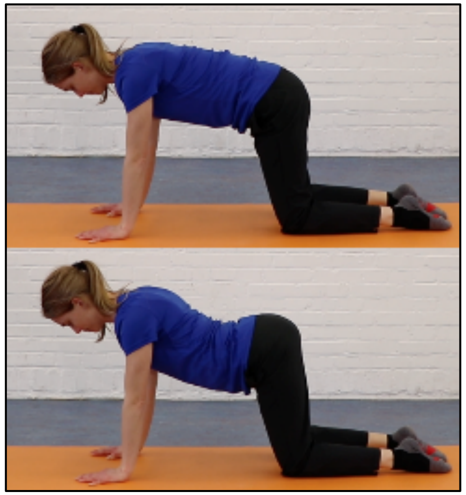

Sitting pelvic tilt
15 repetitions.

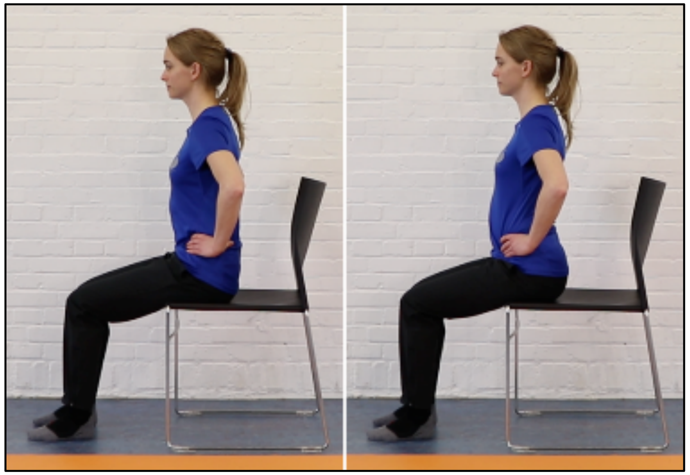

Pelvic tilt on chair
15 repetitions.

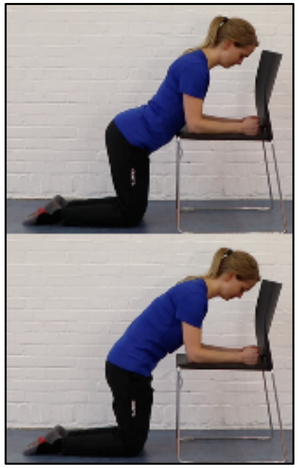

Week 2

Stretching legs
10 repetitions per leg.

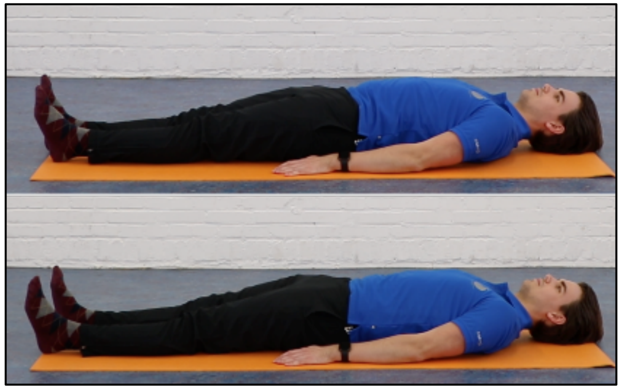

Flexion and extension of the thoracic spine
15 repetitions.

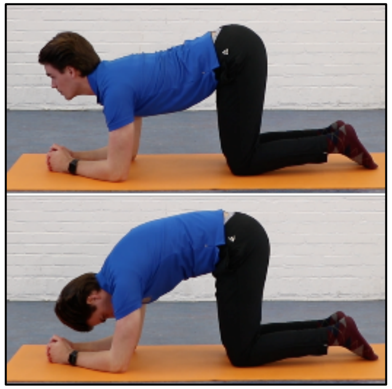

Kneeling pelvic tilt
15 repetitions.

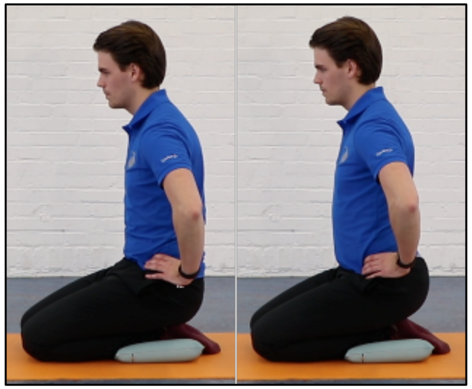

3D rotation of the thoracic spine
10 repetitions per side.

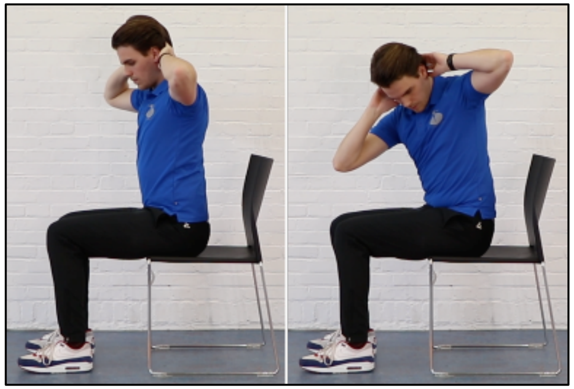

Week 3

Lateral tilt of the lumbar spine
10 repetitions per side.

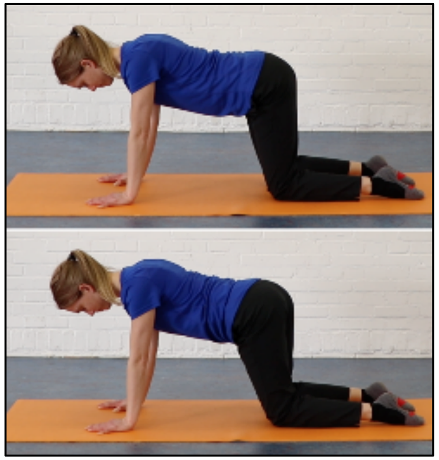

Standing pelvic tilt
15 repetitions.

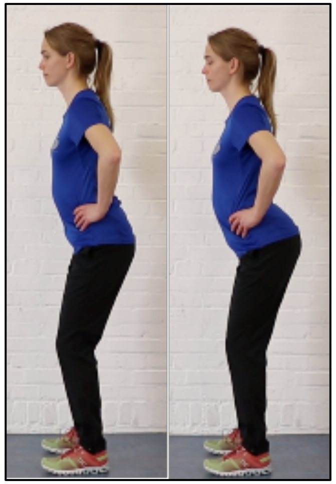

Windshield wipers with feet on the floor
10 repetitions per side.

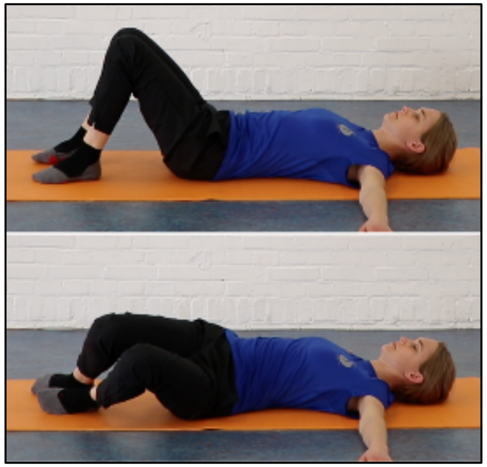

Leaning pelvic tilt
15 repetitions.

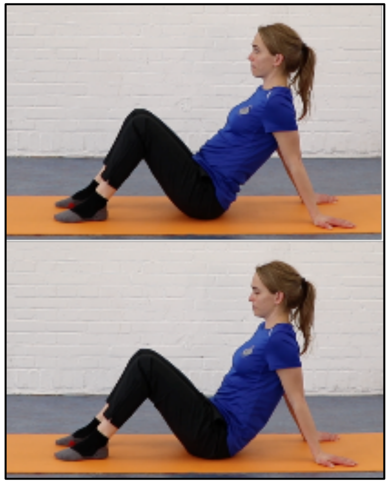

Week 4

Lateral bending
10 repetitions per side.

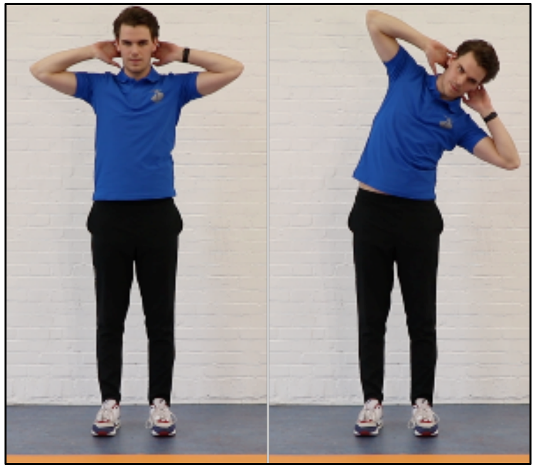

Leg raise with rotation
10 repetitions per leg.

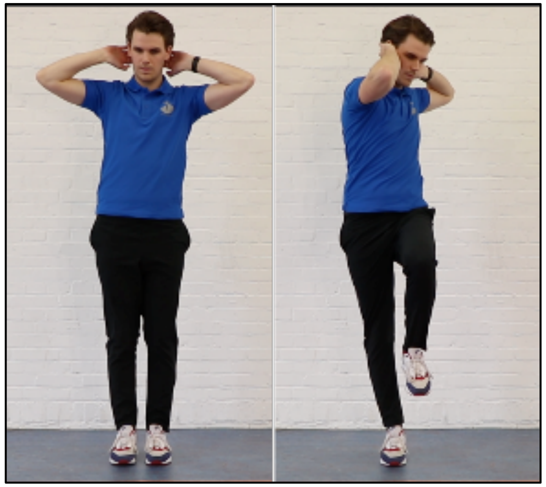

Hands to knees
15 repetitions.

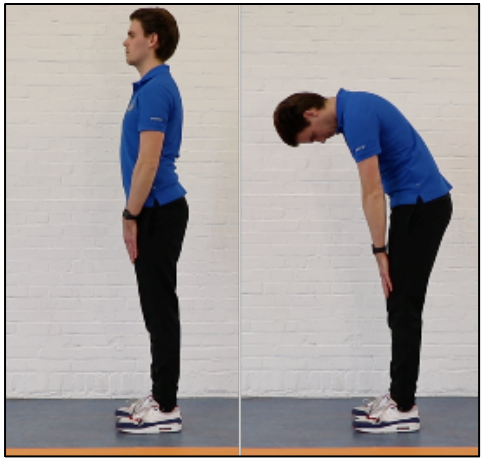

Kneeling pelvic tilt with rotation
10 repetitions.

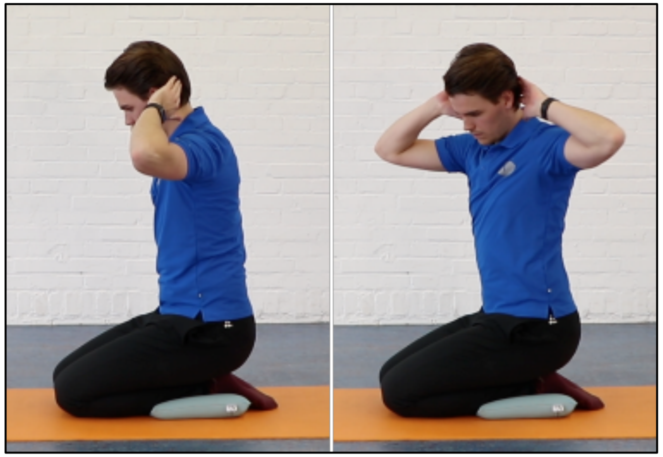

Week 5

Hula hoop in crawling position
5 repetitions per side for 2 times.

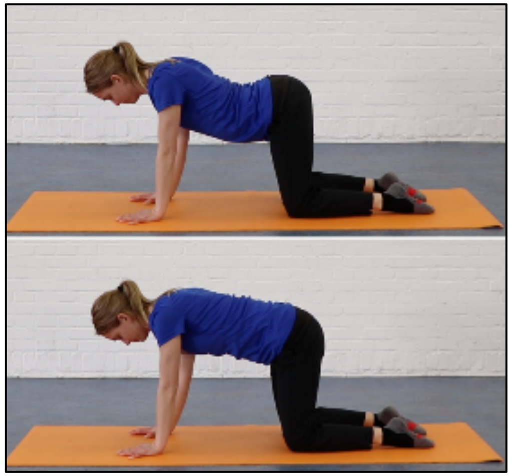

Standing rotation in flexion
15 repetitions per side.

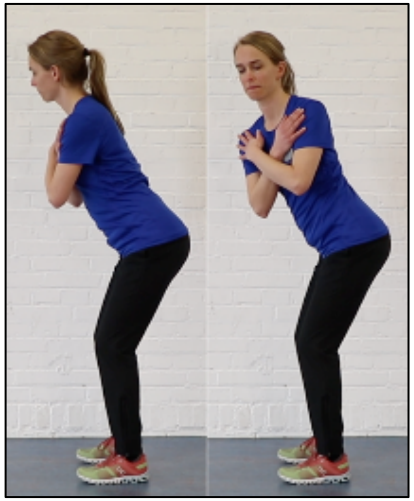

Pelvic tilt in lifted bridge position
10 repetitions.

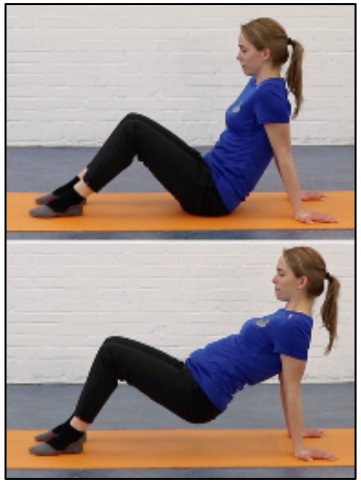

Circling with bent knees
5 repetitions per side for 2 times.

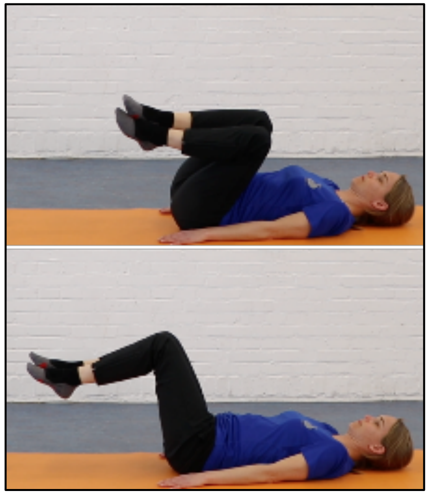

Week 6

Side step lateral bending

8 repetitions per leg

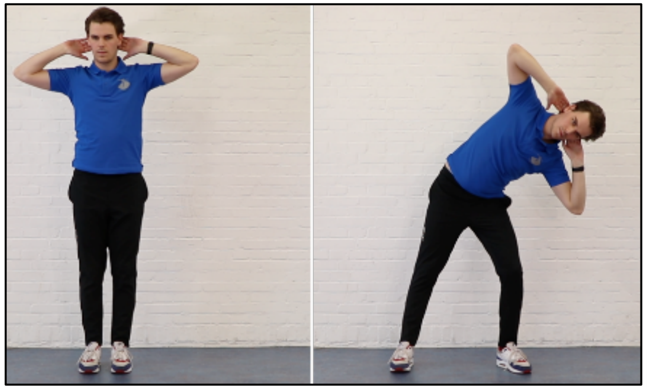

Stepping leg raise with rotation

10 repetitions per side.

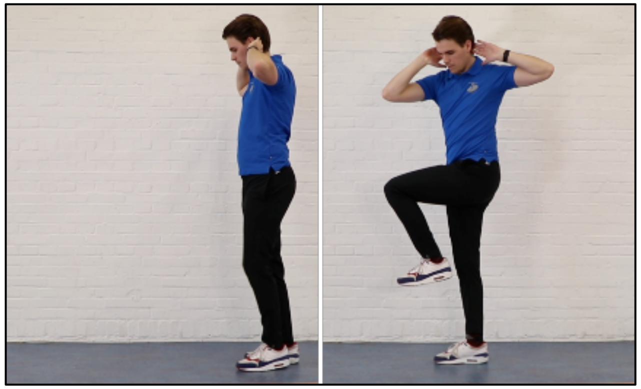

Hands to knees with rotation

7 repetitions per side.

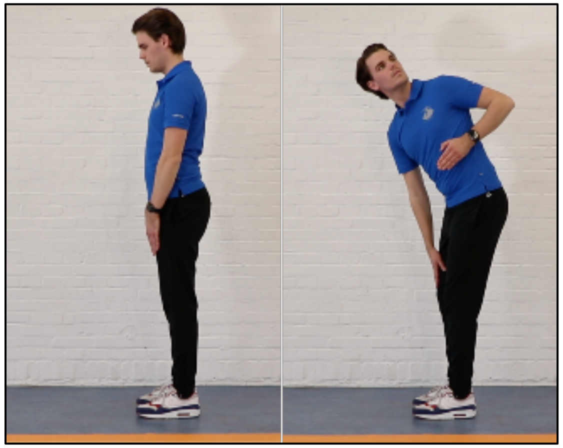

Rotation in kneeling position

4 repetitions per side for 3 times.

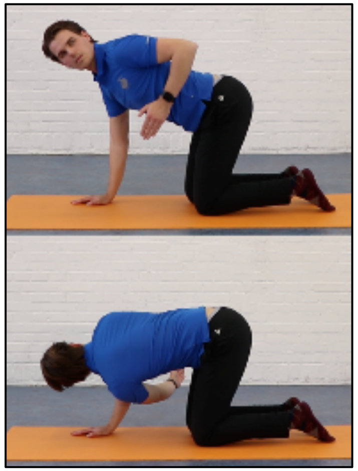

Week 7

Stepping 3D rotation
8 repetitions per side

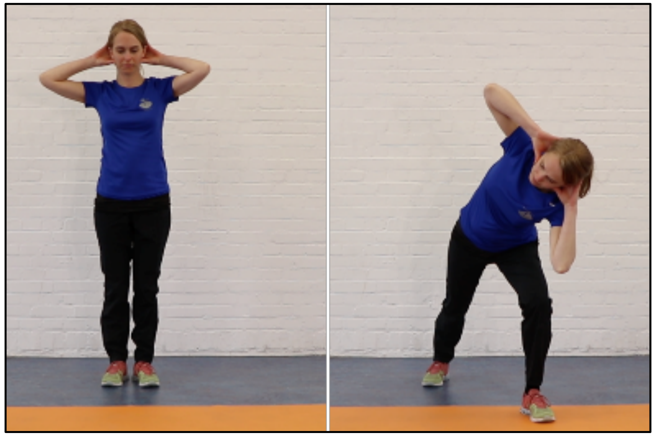

Standing rotation
10 repetitions

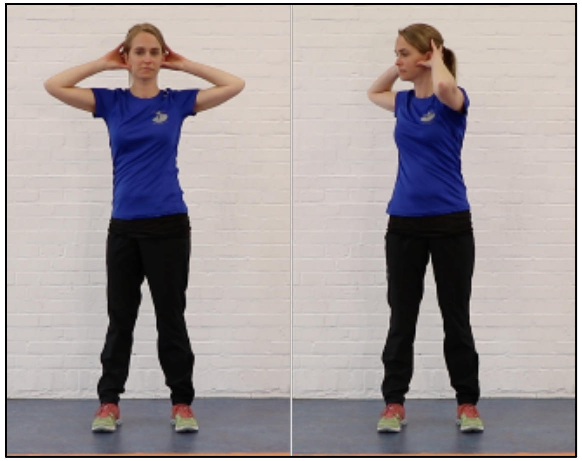

Contrarian rotation
10 repetitions per side

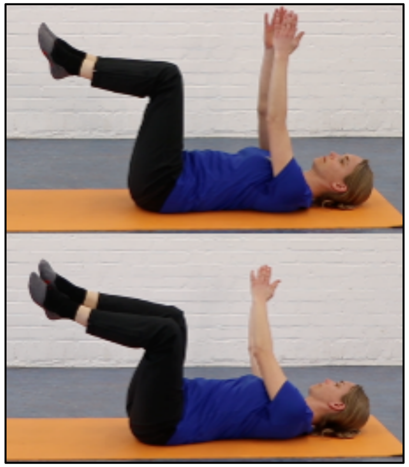

Standing Ichthus
7 repetitions

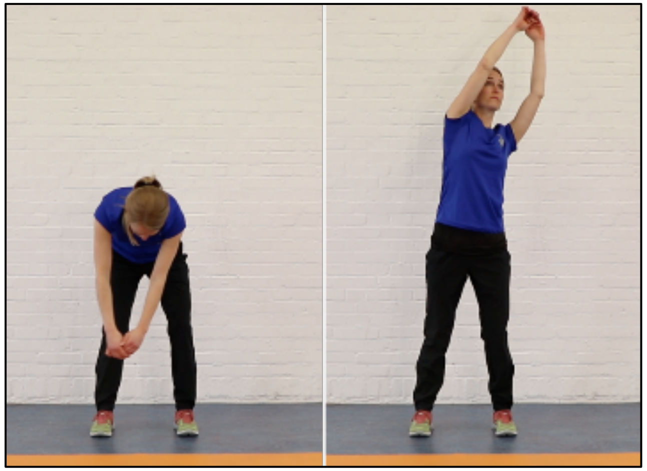

Week 8

Dynamic lunge
10 repetitions per side for 2 times.

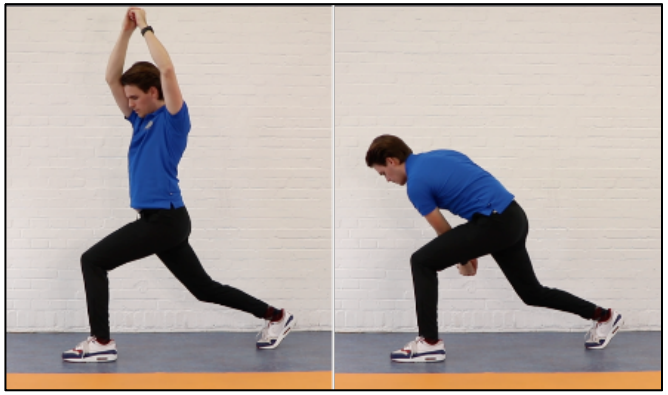

Standing hula hoop
15 repetitions per side.

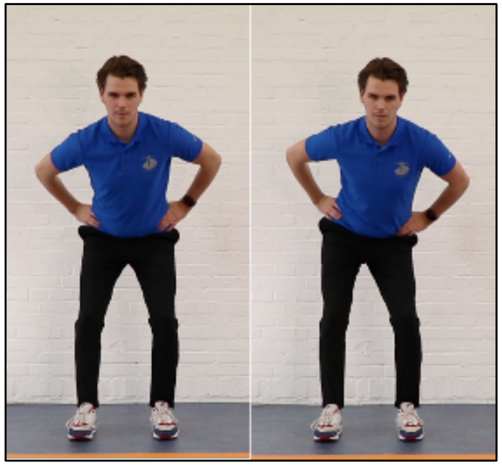

Rotational lunge
5 repetitions per side.

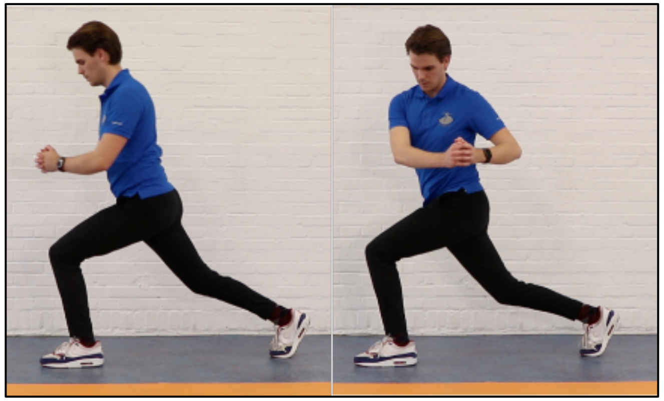

Standing 3D rotation of the thoracic spine
10 repetitions per side

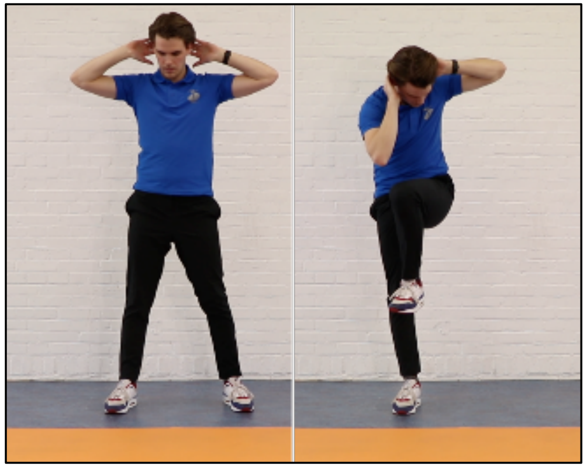

Supplement: Supplementary file 2 [file Data_Sheet_2.pdf]
