# Supplementary material for: Sensor-based intervention to enhance movement control of the spine in low back pain: Protocol for a quasi-randomized controlled trial
Source: Front Sports Act Living. 2022 Oct 17;4:1010054. doi: 10.3389/fspor.2022.1010054 (PMC9619097; doi:10.3389/fspor.2022.1010054)

Supervised and Home exercises Core Stability intervention (week 1 – 8)

Overview of all core stability training exercises used in this study. A full guideline of 4 exercises per week.

Week 1

Tighten TVA exercise

Maintain position for 3 seconds, 15 repetitions.

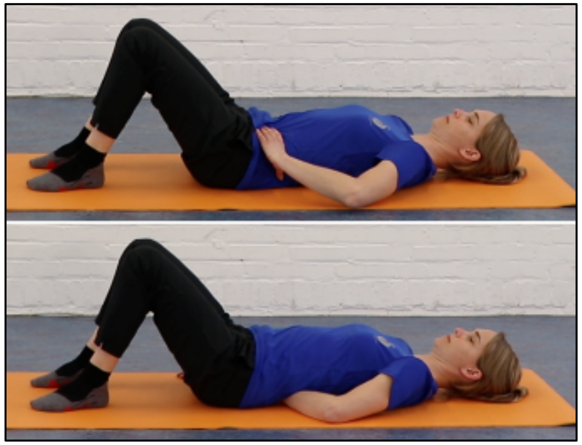

Leg stretch exercise

10 repetitions per leg.

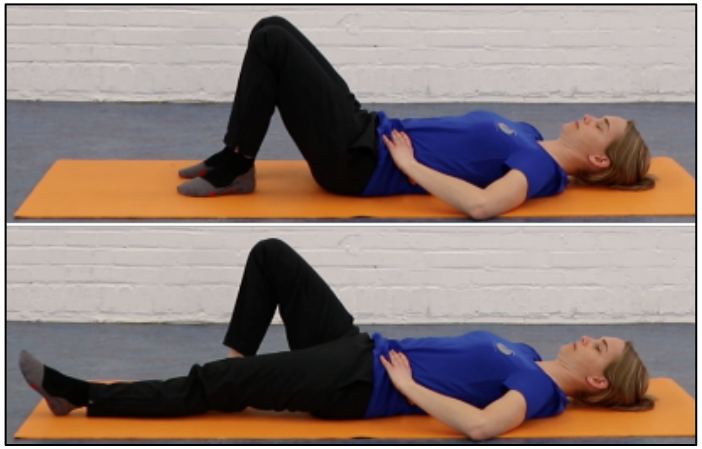

Leg lift exercise

10 repetitions per leg.

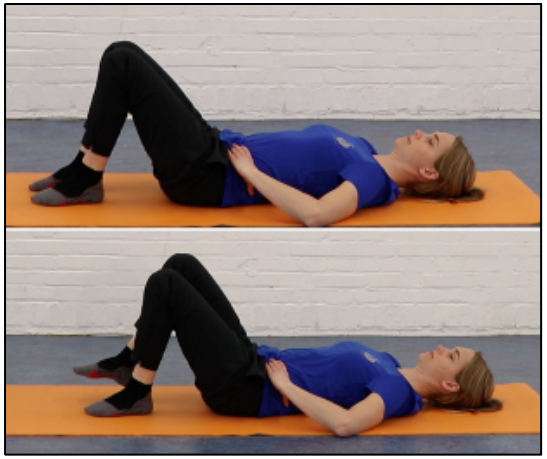

Arm raise exercise

15 repetitions.

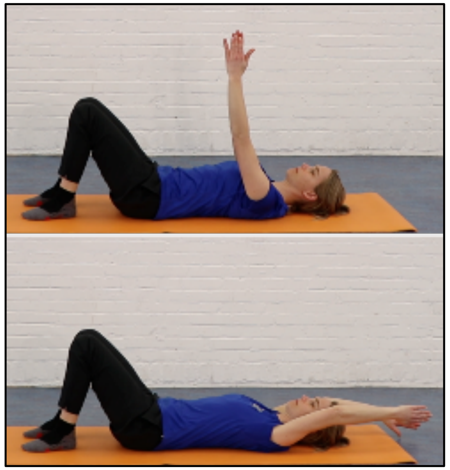

Week 2

Single arm raise exercise
10 repetitions per arm.

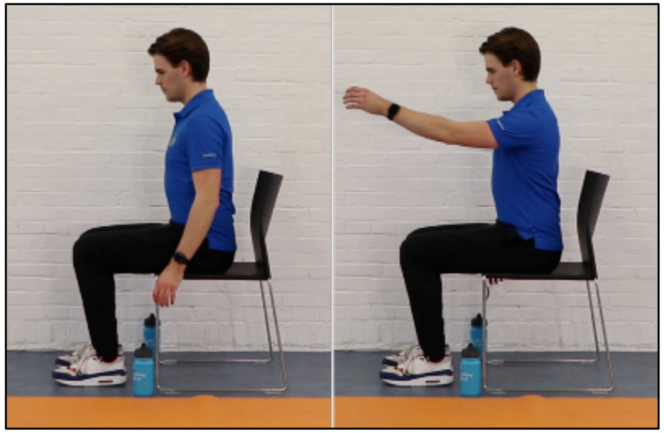

Sitting leg raise exercise
10 repetitions per leg.

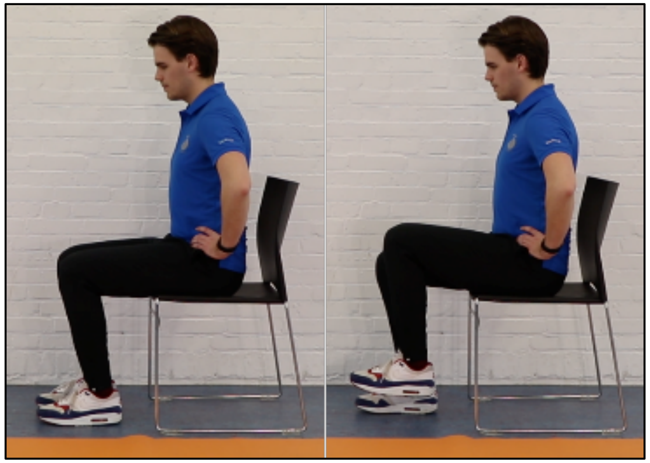

Standing leg raise
10 repetitions per leg.

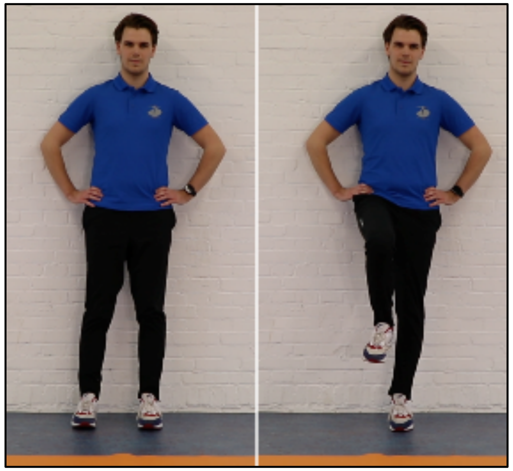

Waiters bow
15 repetitions.

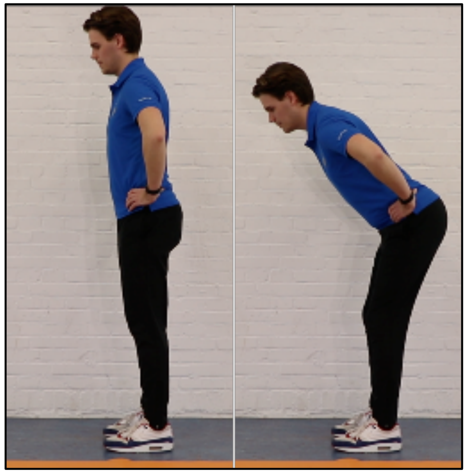

Week 3

Sitting knee push exercise
12 repetitions per side.

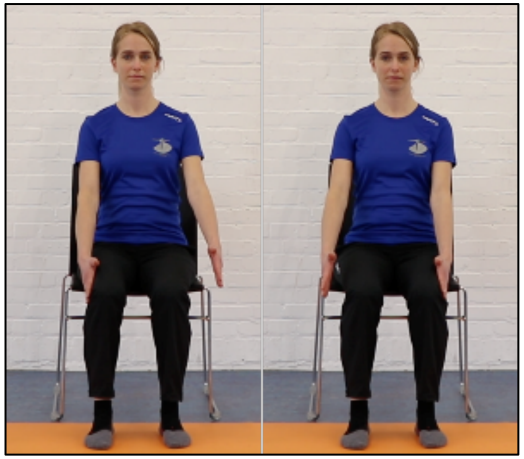

Sidewards leg raise
10 repetitions per leg.

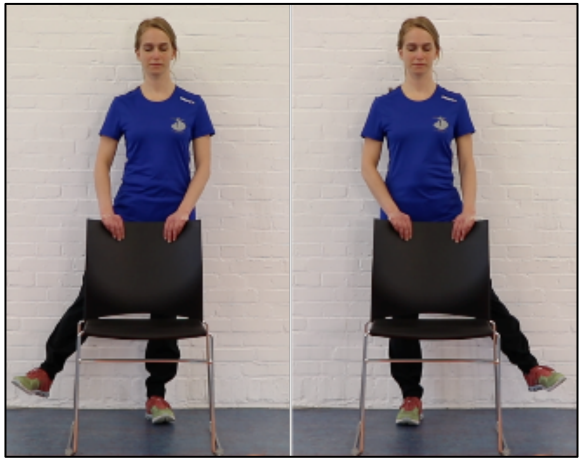

Standing leg raise 2
10 repetitions per leg

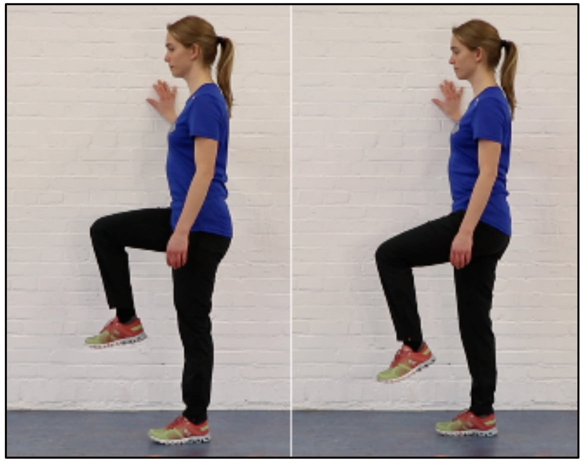

Light squat exercise
15 repetitions.

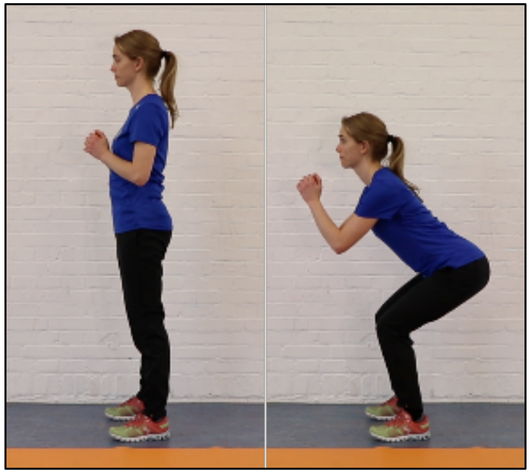

Week 4

Bridge exercise

15 repetitions.

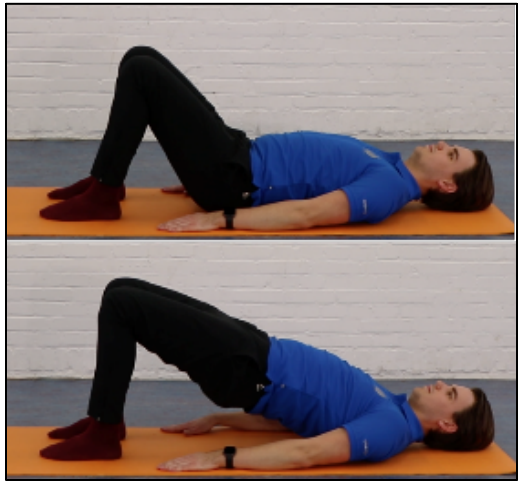

Superman exercise

Movement of arms and legs separately. 5 repetitions per arm and per leg.

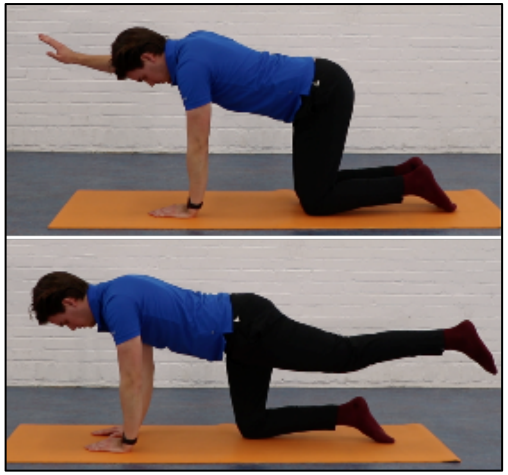

Planking exercise

Maintain position for 5 seconds, 10 repetitions.

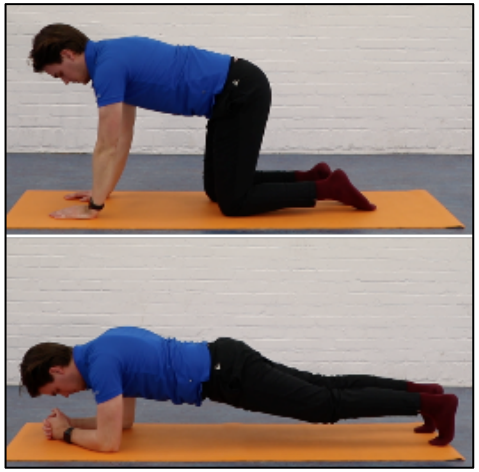

Dynamic knee raise

10 repetitions per leg.

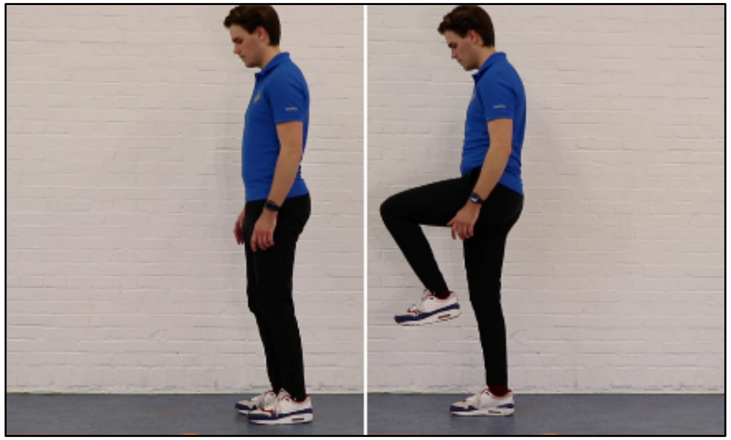

Week 5

Leg stretch 2 exercise

10 repetitions per leg.

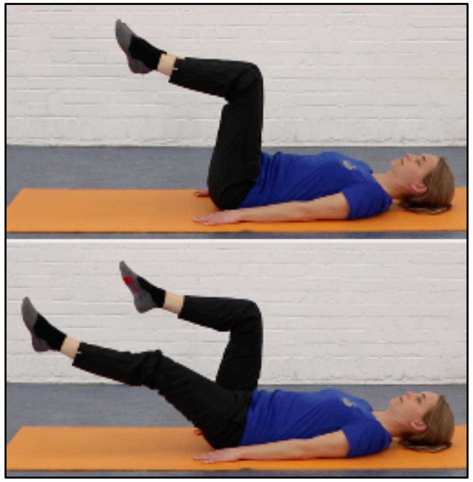

Downward facing dog

Maintain position for 3 seconds,
10 repetitions.

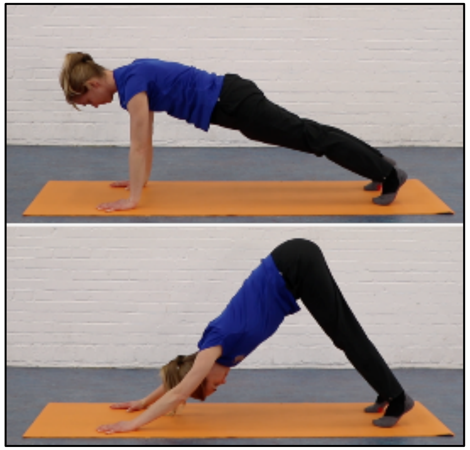

Standing knee push

10 repetitions per leg.

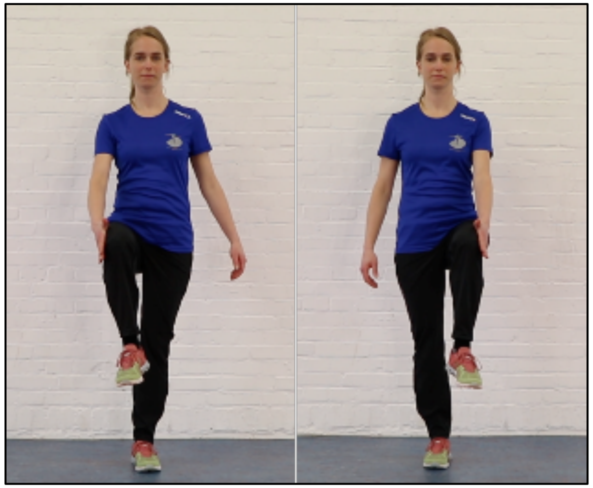

Plank sideways with arm raise

3 repetitions per side for 2 times.

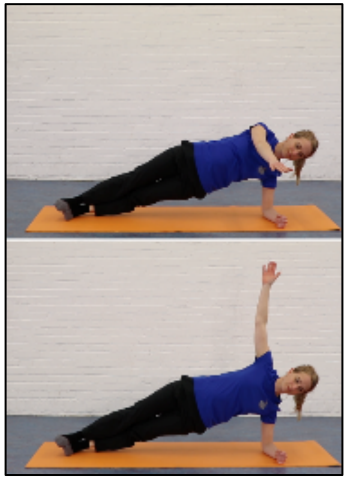

Week 6

Bridge with leg raise

Maintain position for 2 seconds, 6 repetitions per leg.

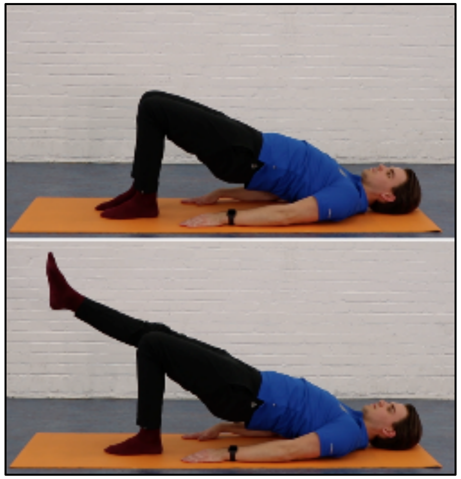

Superman 2

Maintain position for 2 seconds, 7 repetitions per leg.

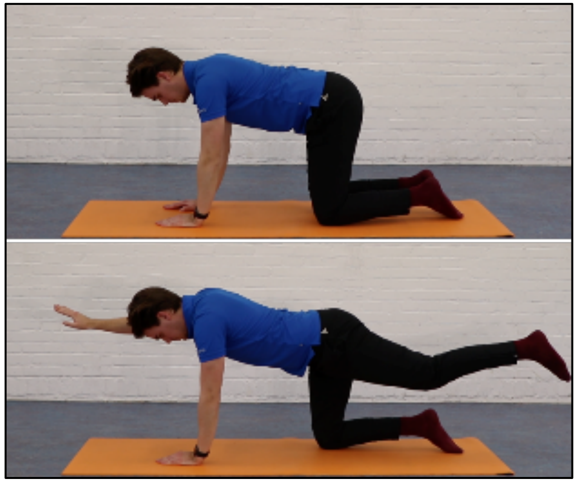

Sidestep knee raise

10 repetitions per leg.

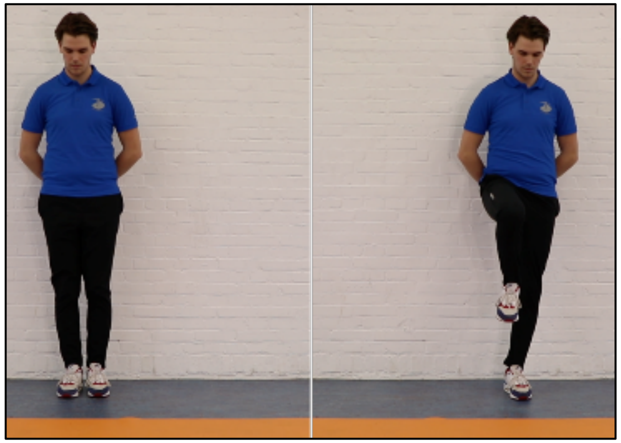

Leg lift with bottle around the body

10 repetitions per leg.

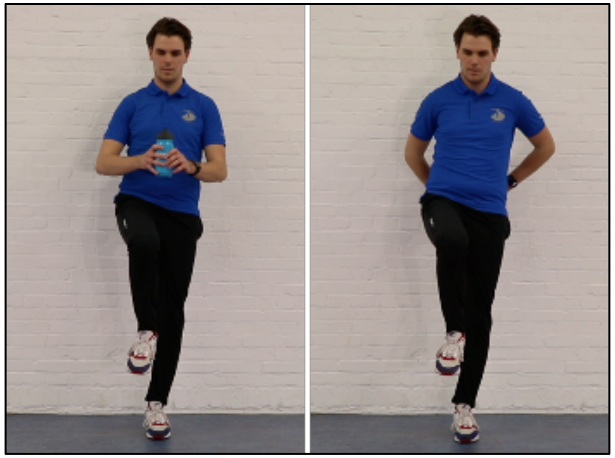

Week 7

Leg raise in circles
2 circles left turn, 2 circles right turn, 3 repetitions per leg.

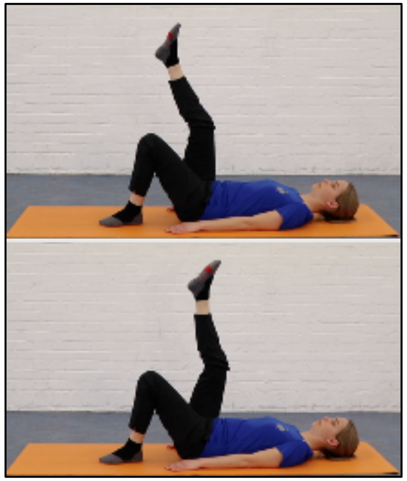

Knee push in forward step
8 repetitions per leg.

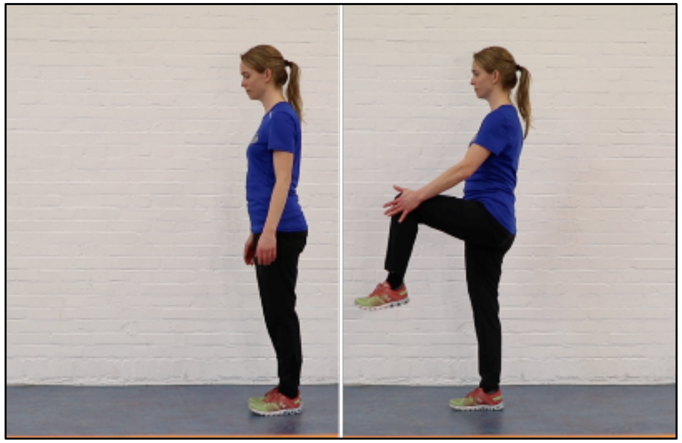

Reverse superman
Maintain position for 2 seconds,
8 repetitions per side.

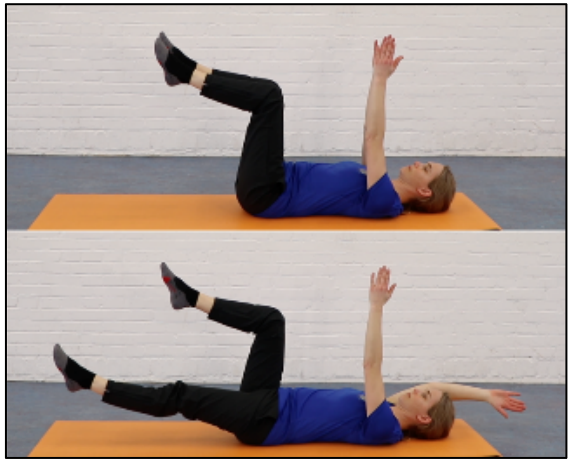

Bridge arm en leg raise
8 repetitions per leg.

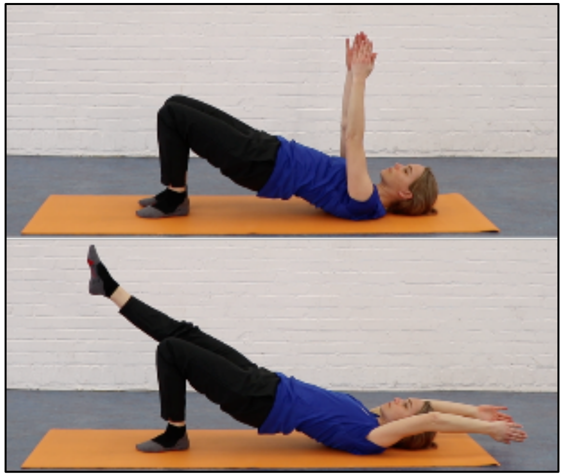

Week 8

Lunge exercise

8 repetitions per leg.

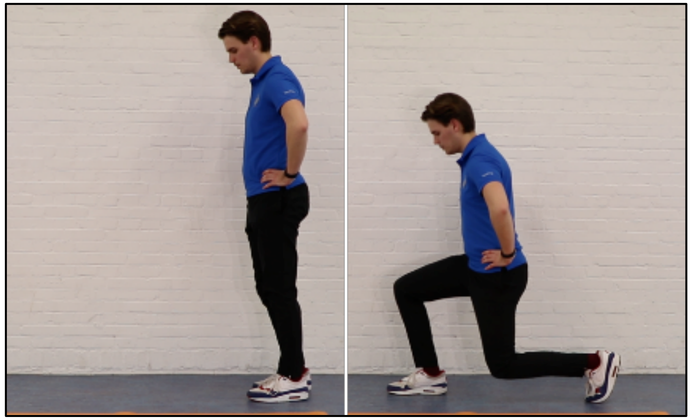

Standing superman

8 repetitions per leg.

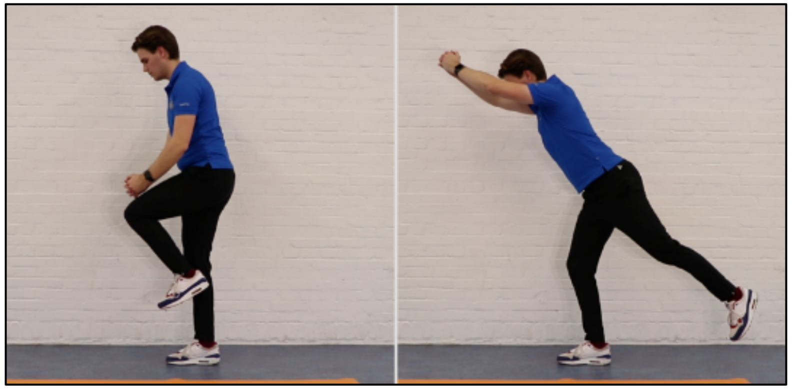

Planking up and down

Maintain position for 2 seconds,
2 repetitions in a row: 4 times.

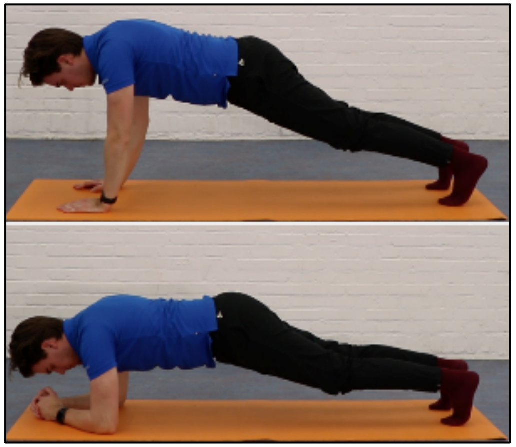

Fluent sidestep knee raise

10 repetitions per leg.

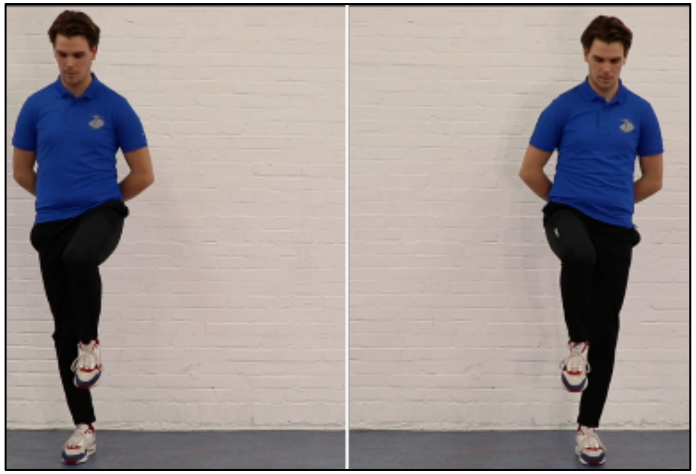

Supplement: Supplementary file 3 [file Data_Sheet_3.pdf]
